# Diel niche variation in mammals associated with expanded trait space

**DOI:** 10.1038/s41467-021-22023-4

**Published:** 2021-03-19

**Authors:** D. T. C. Cox, A. S. Gardner, K. J. Gaston

**Affiliations:** Environment and Sustainability Institute, University of Exeter, Penryn, Cornwall, TR10 9FE UK

**Keywords:** Phenology, Ecosystem ecology, Evolutionary ecology, Macroecology, Taxonomy

## Abstract

Mammalian life shows huge diversity, but most groups remain nocturnal in their activity pattern. A key unresolved question is whether mammal species that have diversified into different diel niches occupy unique regions of functional trait space. For 5,104 extant mammals we show here that daytime-active species (cathemeral or diurnal) evolved trait combinations along different gradients from those of nocturnal and crepuscular species. Hypervolumes of five major functional traits (body mass, litter size, diet, foraging strata, habitat breadth) reveal that 30% of diurnal trait space is unique, compared to 55% of nocturnal trait space. Almost half of trait space (44%) of species with apparently obligate diel niches is shared with those that can switch, suggesting that more species than currently realised may be somewhat flexible in their activity patterns. Increasingly, conservation measures have focused on protecting functionally unique species; for mammals, protecting functional distinctiveness requires a focus across diel niches.

## Abstract (web summary)

Most mammals are nocturnal, but a new analysis suggests that although most groups of species active at a particular time of day or night occupy different ecological niches, a surprisingly large proportion of species are more flexible in the timing of their activity than previously thought.

## Introduction

In the Mesozoic, most mammals were restricted to nocturnal activity to avoid antagonistic interactions with the ecologically dominant diurnal dinosaurs (‘nocturnal bottleneck’)^1,2^. After the extinction of all non-avian dinosaurs circa 66 million years ago, those surviving mammal species experienced rapid diversification in body masses, modes of locomotion, diets and activity patterns^1,2^. However, although mammals now exhibit strikingly varied morphological, behavioural and ecological niches^3^, activity patterns are still strongly biased towards nocturnality^4^.

The diel activity pattern of a species will determine the biotic (e.g. predation, competition) and abiotic (e.g. light levels, temperature) regimes to which it is exposed, and species have evolved suites of functional traits to reflect these pressures and to maximise fitness during times of activity^5,6^. As such, when species are active may lead to convergent combinations of traits that distinguish them from species that exploit different diel niches (e.g. ectothermy and endothermy in lizards^7^). These trait combinations reflect the spatiotemporal distribution of resource capture, utilisation and release and so determine the species’ ecological role, its contribution to ecosystem functioning^8^ and its responses to environmental change^9,10^. Thus, they can be seen to summarise a species’ ecological strategy^11,12^.

Functional trait hypervolumes characterise phenotypic space occupied by a set of species^13^. Quantifying the volume and overlap of multidimensional functional trait space enables inferences about how differing ecological and evolutionary processes structure functional diversity and ecological strategies^8,14^. As yet, it is unclear whether mammal activity patterns manifest in the occupation of unique regions of functional trait space, thereby reflecting emphasis on different ecological strategies.

Although mammals tend to have a dominant diel strategy, many species show flexibility in the times when they are active^6,15,16^, switching their diel niche in response to factors, such as predictable changes in dietary composition^17^, removal of predation^18^, or in the face of novel environmental, climatic or anthropogenic pressures^14,18^. Those species that can change the timing of their activity will be faced with different abiotic and biotic pressures, which may have shaped the evolution of their traits^19^. However, it is unknown whether the ability of species to be active outside of their dominant diel niche requires specific trait combinations, or whether most mammals possess similar traits that might give them the ability to switch the timing of their activity in the face of ecological and environmental pressures.

Here we explore three primary research questions: (1) do the major gradients of ecological strategies differ between mammal species with different diel niches? (2) do species exploiting different diel niches occupy unique regions of functional trait space? (3) do species with flexible activity patterns exhibit certain combinations of functional traits? For 5104 extant mammal species, we assigned each as being nocturnal, crepuscular, cathemeral or diurnal. We then collated information on five major functional traits: body mass, litter size, diet, foraging strata and habitat breadth (Supplementary Methods 1). For each diel niche, we (1) ordinated functional traits to represent a two-dimensional continuum that forms an ecological strategy surface, allowing comparison of major gradients of species. We then constructed a five-dimensional trait hypervolume to assess through comparative analyses how trait combinations are shared (2) across different diel niches, and (3) between diel flexible and diel obligate species.

Here, we show that species that are active during the daytime, either partly (cathemeral) or fully (diurnal), evolved different gradients of trait combinations from those active during the nighttime (nocturnal or crepuscular). Hypervolume analysis reveals that over half of nocturnal trait space, and almost a third of diurnal trait space is unique demonstrating that these strategies have not been replicated in different diel niches and species active during these times may affect ecosystem processes and functions differently. Moreover, diel flexible and diel obligate hypervolumes show that most species that can switch the timing of their activity share trait space with species not recorded as doing so, suggesting that the ability to be somewhat flexible in the timing of activity may be more universal than currently thought. However, over half of the diel obligate hypervolume is unique revealing that many species may be unable to switch the timing of their activity in the face of anthropogenic change.

## Results

We found that 70.1% of mammals are primarily nocturnal (3580 species), compared to 2.5% (126 species) that are crepuscular, 9.1% (467 species) cathemeral and 18.2% (931 species) diurnal. There was phylogenetic conservatism in time-partitioning strategy within mammals as a whole (Pagel’s *λ* = 0.955, 0.004 standard deviations (SD)), and within the individual diel niches (nocturnal Pagel’s *λ* = 0.965, 0.003 SD; crepuscular Pagel’s *λ* = 0.911, 0.01 SD; cathemeral Pagel’s *λ* = 0.936, 0.008 SD; diurnal Pagel’s *λ* = 0.963, 0.004 SD; where SD is the standard deviation of Pagel’s *λ* calculated across 30 randomly selected phylogenetic trees).

### Ecological strategy surfaces and diel niches

**Fig. 1:**
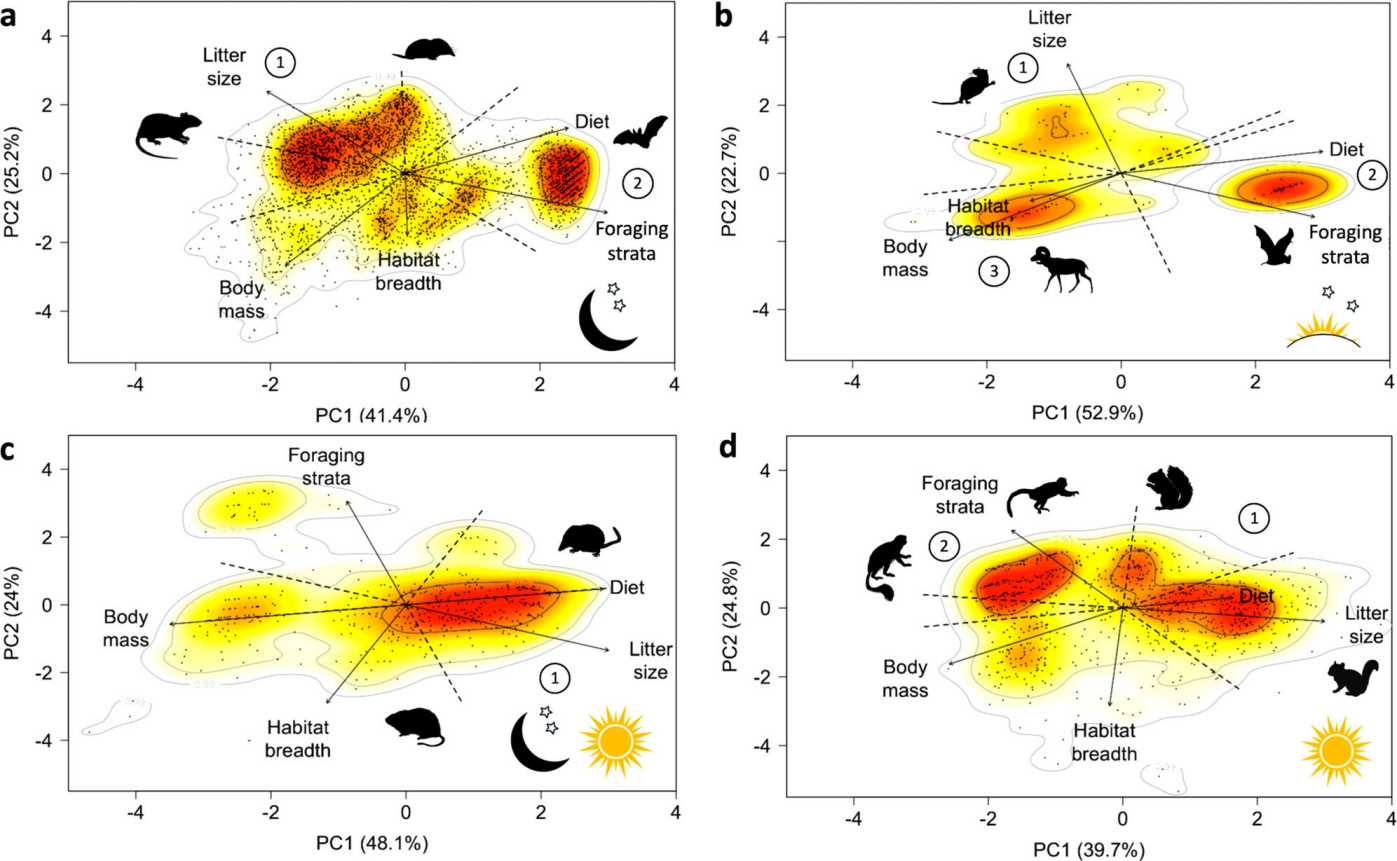
Ecological strategy surfaces associated with mammals occupying different diel niches. **a** Nocturnal (*n* = 3580, moon and stars silhouette), **b** crepuscular (*n* = 126, sunrise/sunset and stars image), **c** cathemeral (*n* = 467, moon, stars and sun image) and **d** diurnal (*n* = 931, sun image). For each diel niche, projections show the species (dots) on the surface defined by principal component axes (PC) 1 and 2. Solid arrows indicate the direction and weighting of vectors representing the five continuous traits analysed, and thus represent the major gradient of each trait (Supplementary Tables 1 and 2 for trait variance and loadings). Percentage values represent the proportion of the total variation explained by each PC. The colour gradient specifies regions of highest (red) to lowest (white) occurrence probability of species across the ecological strategy, with contour lines indicating 0.5, 0.95 and 0.99 quantiles. Thus, red regions correspond to functional hotspots and circled numbers denote the hotspots in each diel niche, as described in the main text. Silhouettes represent species characterising the hotspots. Large hotspots display two silhouettes, representing species at the hotspot extremes (silhouettes were freely downloaded from PhyloPic www.phylopic.org, under CC0 1.0 Public Domain Dedication and from Adobe Stock Images under Standard License).

Despite high diversity in functional trait combinations across different diel niches, nocturnal and crepuscular species converged on a different ecological strategy from species that use the daytime either partly (cathemeral) or solely (diurnal). This is shown by the similarity in the distribution of major trait gradients across the first two principal component axes in nocturnal and crepuscular species and in cathemeral and diurnal species, and also by the different relative directions of the major trait gradients along the first two principal component axes between night and day-active species (Fig. 1; note that when treated independently the directions of each trait in ordinated trait space are arbitrary). For each diel niche, the first two principal components (PC1 and PC2) accounted for approximately two-thirds of the total trait variation, with body mass, litter size and foraging strata explaining the most variance across all diel niches (Supplementary Table 1). There were clear functional hotspots—areas of particularly dense species occupation—in trait space, and these can be thought to contain species that typify that diel niche (Fig. 1).

For nocturnal and crepuscular species, the primary axis of differentiation (PC1) integrates gradients of body mass (small to large, loading −0.41 for nocturnal and −0.53 for crepuscular species), diet (herbivore to insectivore; loading 0.50 for nocturnal and 0.55 for crepuscular species) and foraging stratum (ground to aerial, loading 0.61 for nocturnal and 0.55 for crepuscular species). The secondary axis of differentiation (PC2) integrates litter size (large to small, loading 0.54 for nocturnal and 0.86 for crepuscular species) and, for nocturnal species only, habitat breadth (specialist to generalist, loading −0.45). Nocturnal species contained two, and crepuscular species three, functional hotspots. The first hotspot for both diel niches crosses mid to low PC1 and PC2 values, and characterises species favouring a more r-selected strategy, having small body masses, large litter sizes, foraging on the ground and being habitat specialists (*n* = 1200 nocturnal species, e.g. the Long-haired rat *Rattus villosissimus*; *n* = 3 crepuscular species, e.g. Elias’s spiny rat *Trinomys eliasai*; Fig. 1a); hotspots capture areas of dense clusters of 50% of species in each diel niche and although the crepuscular hotspot only contained three species, its inclusion facilitates comparisons across diel niches. The second hotspot for both diel niches is clustered around high PC1 values and corresponds to aerial foraging insectivorous bats (*n* = 591 nocturnal species, e.g. Trident leaf-nosed bat *Aselliscus tricuspidatus*; *n* = 3 crepuscular species, e.g. Northern ghost bat *Diclidurus albus;* Fig. 1a, b). The third crepuscular hotspot represents more k-selected species with large body mass, an herbivorous diet, generalist habitat breadth and that forage on the ground (*n* = 29 species; e.g. White-tailed Deer *Odocoileus virginianus*; Fig. 1b).

For cathemeral and diurnal species PC1 integrates gradients of body mass (small to large, loading −0.60 and −0.57, respectively), litter size (large to small, loading 0.63 for cathemeral and 0.53 for diurnal species) and diet (insectivore to herbivore, loading 0.50 for cathemeral and 0.34 for diurnal species) and PC2 integrates foraging strata (ground to aerial, loading 0.54 for cathemeral and 0.74 for diurnal species) and habitat breadth (generalist to specialist, loading −0.65 for cathemeral and −0.75 for diurnal species). Cathemeral species have one functional hotspot, whilst diurnal species display two. The first and largest hotspot shared by both cathemeral and diurnal species in its approximate position on the axes of differentiation characterises species favouring a more r-selected strategy (relatively small body mass and large litter sizes, and a herbivorous to carnivorous diet (*n* = 234 cathemeral species and 267 diurnal species; e.g. small rodents, such as the cathemeral Forest grass mouse *Akodon torques*, and the diurnal Common treeshrew *Tupaia glis*; Fig. 1c, d). The second diurnal hotspot, formed around low PC1 values, distinguishes species of medium body mass, small litter sizes, arboreal foraging and having an herbivorous diet (*n* = 191 species; i.e. primates, for example, Black howler monkey *Alouatta nigerrima*; Fig. 1d) and represents a more k-selected strategy.

The first hotspot for nocturnal, cathemeral and diurnal species consists of low-level foraging rodents and their allies, and we find marked differences between diel niches in body mass, litter sizes and diet (Fig. 1a, c, d; hotspots 1). Nocturnal species in the first hotspot converged on smaller body masses (*n* = 1200; median 200 g, interquartile range (IQR): 101 g, 406 g) and litter sizes (median 3.6; IQR: 3, 4.1), and an herbivorous diet. Conversely, species in the cathemeral hotspot maintain low body masses (*n* = 234; median body mass 27 g, IQR: 9 g, 48 g), large litter sizes (median 4.1, IQR: 3.4, 5.2) and have an omnivorous or carnivorous diet. Diurnal species in the first hotspot have larger body masses (*n* = 267; median 213 g, IQR: 108 g, 408 g), relatively small litter sizes (median 3.2; IQR: 2, 4.1) and a range of herbivorous to carnivorous diets. The second hotspot for nocturnal and crepuscular species contains aerial insectivorous bats, and there was high convergence between the two hotspots in species traits (*n* = 591 nocturnal species; e.g. median body mass 27 g, IQR: 9 g, 48 g; *n* = 32 crepuscular species, e.g. median body mass 6.3 g, IQR: 5.2 g, 10.8 g).

Ecological strategy surfaces showed that the least number of families was captured by the two nocturnal hotspots (43% of families), conversely the diurnal strategy surface also displayed two hotspots but captured the greatest number of families (66% of families; Fig. 1; Supplementary Data 1). The three crepuscular hotspots captured a greater number of families (capturing 65% of families) than the single cathemeral hotspot (capturing 46% of families; Supplementary Data 1).

### Functional trait space between diel niches

**Fig. 2:**
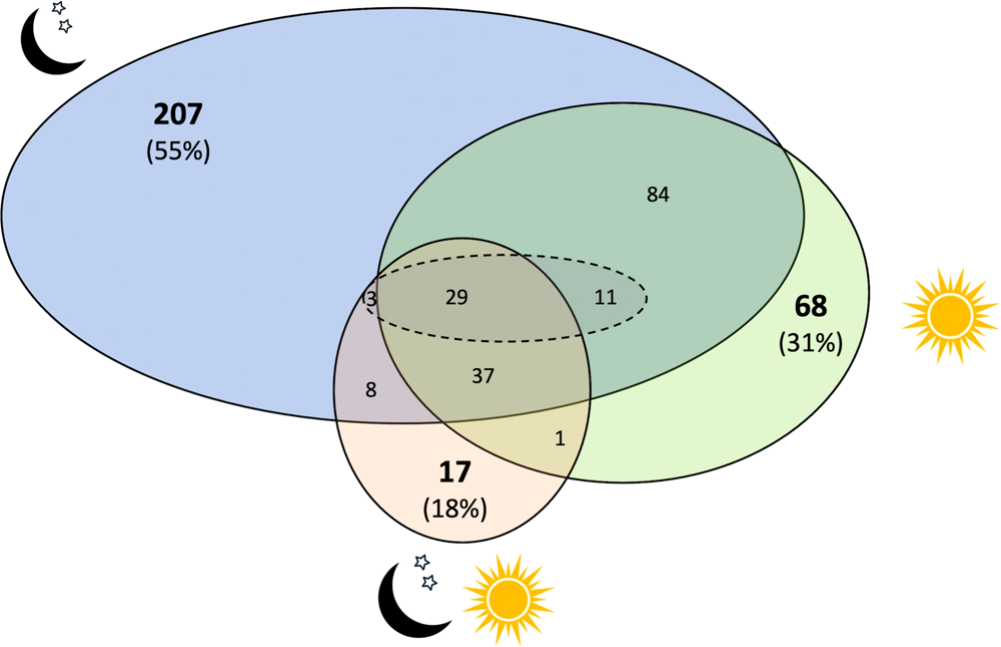
Two-dimensional representation of the overlap in five-dimensional trait space of species occupying different diel niches. Nocturnal species are shown in blue (full volume 375 SD^5^, moon and stars silhouette), crepuscular species by the dash oval (full volume 43 SD^5^), cathemeral species in peach (full volume 95 SD^5^, moon, stars and sun image) and diurnal species in green (full volume 226 SD^5^, sun image). Hypervolumes for each diel niche were constructed on the basis of five z-transformed traits: body mass (log_10_), litter size (log_10_), diet, foraging strata and habitat breadth (square root transformed). Comparative analyses on paired hypervolumes were carried out, where numbers in bold specify the unique volume for each diel niche and numbers not in bold give the overlapping volumes of diel niches. The percentage of the hypervolume that is unique to each diel niche is given in parentheses. Statistical approaches are not available for comparing more than two hypervolumes, and so overlapping volumes are estimated based on paired overlaps (See Supplementary Table 3 for paired hypervolume analyses with unmatched sample sizes). The units of the unique and overlapping fractions are SD^5^. Note that one crepuscular species had a unique combination of traits that was not possible to represent in the figure.

There was significant variation in trait hypervolume size between the four diel niches that corresponded to the number of species using that diel niche (the nocturnal hypervolume was the largest, 375 SD^5^; and the crepuscular hypervolume the smallest, 43 SD^5^; where hypervolume units are the SD of trait values, raised to the power of the number of dimensions (5)). Comparative paired analysis of each diel niche hypervolume with all species using alternative diel niches revealed that 55% of the nocturnal hypervolume is unique to species that are active at night (Table 1; Fig. 2). Conversely, only 17% of the non-nocturnal hypervolume was unique (Table 1a; Fig. 2). More than 99% of the crepuscular hypervolume overlaps with the nocturnal, and to a slightly lesser extent the diurnal hypervolume (92%; Supplementary Table 3a; Fig. 2). Only 18% of the cathemeral hypervolume was unique, and the 82% that was not unique only overlapped with a total of 21% of the combined hypervolumes of the other activity patterns. Over a quarter of the diurnal hypervolume was uniquely diurnal (30%); for these species, activity solely during the daytime has required the evolution of unique trait combinations.

**Table 1:** Overlap in hypervolume estimation between mammals occupying different diel niches. Comparative paired analysis of each diel niche was carried out compared to (a) all species with other diel niches, and (b) species with each of the other diel niches in turn.

Hypervolume 1 (H1)	Hypervolume 2 (H2)	Volume H1 (SD^5^)	Volume H2 (SD^5^)	Unique fraction H1	Unique fraction H2
(a) Comparison with all species					
Nocturnal	Excluding nocturnal	375	206	0.55	0.17
Crepuscular	Excluding crepuscular	43	368	0.01	0.89
Cathemeral	Excluding cathemeral	96	379	0.18	0.79
Diurnal	Excluding diurnal	226	359	0.30	0.56
(b) Comparison with other diel niches^a^					
Nocturnal (126)	Crepuscular	85 (±14)	43	0.59 (±0.05)	0.21 (±0.06)
Nocturnal (467)	Cathemeral	184 (±14)	95	0.66 (±0.02)	0.34 (±0.04)
Nocturnal (931)	Diurnal	247 (±15)	226	0.43 (±0.02)	0.38 (±0.02)
Cathemeral (126)	Crepuscular	33 (±9)	43	0.46 (±0.07)	0.63 (±0.09)
Diurnal (126)	Crepuscular	77 (±13)	43	0.61 (±0.04)	0.31 (±0.07)
Diurnal (467)	Cathemeral	170 (±13)	95	0.62 (±0.02)	0.34 (±0.03)

Hypervolumes were constructed using the five z-transformed traits: body mass (log10), litter size (log10), diet, foraging strata and habitat breadth (square root transformed). To control for differences in sample sizes between comparisons of individual diel niches, the number of species in the larger volume was matched with the smaller volume. The numbers of randomly sampled species in the reduced larger hypervolume are given in parentheses. Hypervolumes were generated from 100 randomly selected subsets of the larger diel niche and the mean volume and mean unique fraction of each hypervolume are presented, the standard deviation is given in parentheses. Hypervolume units are the standard deviations of trait values, raised to the power of the number of dimensions (SD^5^).

^a^Randomly sampled matched subset with smaller hypervolume.

We carried out comparative paired analysis of each diel niche hypervolume, against each of the other diel niche hypervolumes in turn. After matching the sample size of the larger hypervolume with that of the smaller one (to control for potential confounding effects of different sample sizes^20^), in each analysis, the nocturnal hypervolume remained the largest and the diurnal hypervolume the second largest (Table 1b). The matched cathemeral hypervolume was smaller than the crepuscular hypervolume (Table 1b). Cathemerality overlapped equally with the matched nocturnal and matched diurnal hypervolumes (Table 1b).

### Functional trait space and mammalian diel flexibility

**Fig. 3:**
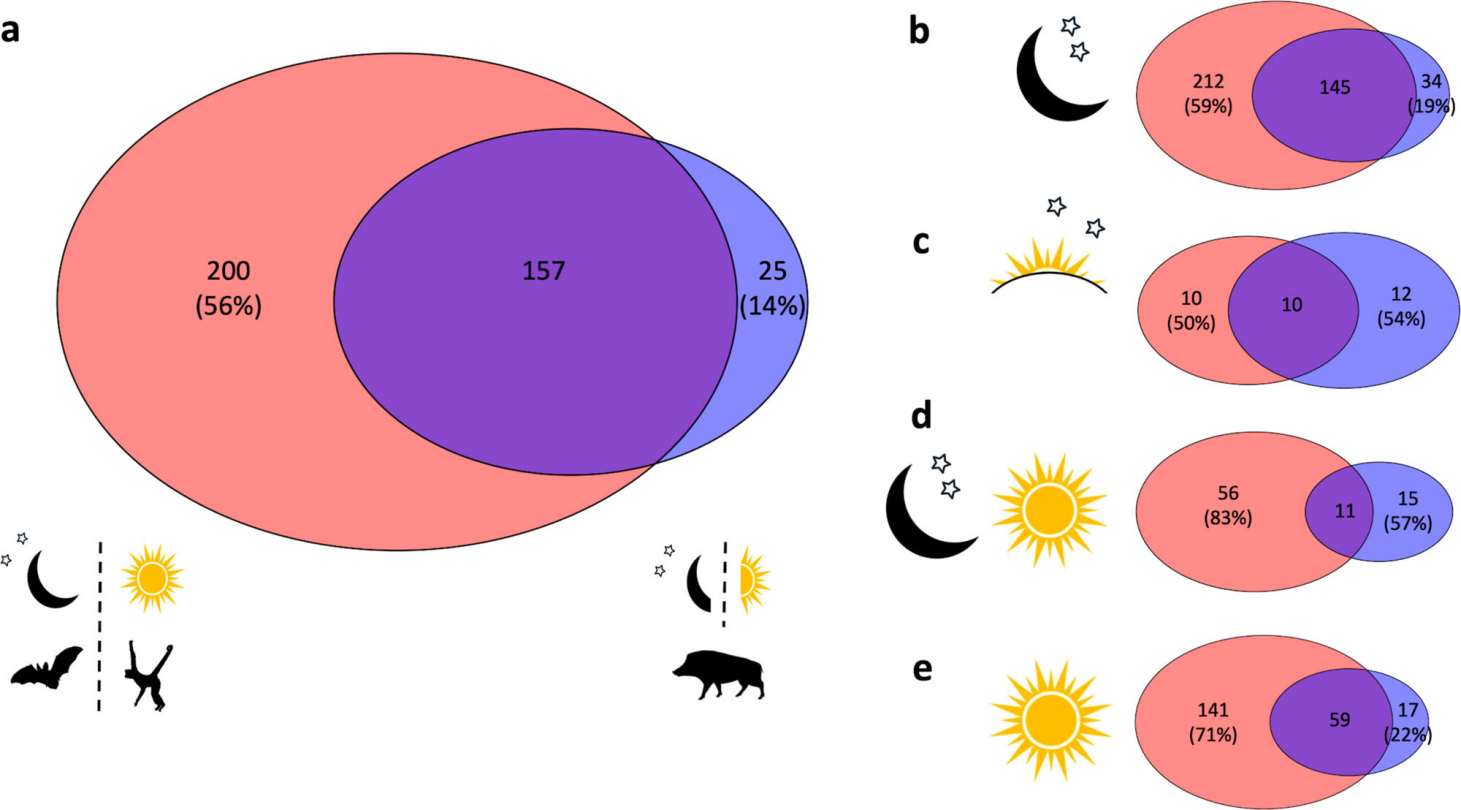
Diel flexibility in functional trait space. Hypervolumes were constructed of diel obligate (red) and diel flexible (blue) species, before assessing comparative statistics across **a** all species, **b** nocturnal species only (moon and stars silhouette), **c** crepuscular species only (sunset/sunrise and stars image), **d** cathemeral species only (moon, stars and sun image) and **e** diurnal species only (sun image). Numbers give the volume of the unique diel obligate hypervolume (red), the unique diel flexible hypervolume (blue) and the overlapping volume between hypervolumes (purple; Supplementary Table 3). The percentage of the hypervolume that is unique to obligate and flexible species is given in parentheses. Mammal silhouettes give examples of diel obligate and diel flexible species and were freely downloaded from PhyloPic www.phylopic.org, under CC0 1.0 Public Domain Dedication.

We found evidence that 18.0% of mammal species have been recorded as being active outside of their dominant diel niche (nocturnal, 592 (16.5%) species; crepuscular, 68 (54.0%) species; cathemeral, 84 (18.0%) species; diurnal, 175 (18.8%) species). Functional hypervolume analysis revealed that 86% of the hypervolume of these species overlapped with the hypervolume of species with apparently more obligate diel niches (Fig. 3a). From the reciprocal perspective, 44% of the diel obligate hypervolume overlapped with the diel flexible hypervolume, whereas 56% of the hypervolume of species with obligate diel niches was unique (Fig. 3a).

Almost two-thirds of the hypervolume of obligately nocturnal species was unique (Fig. 3b). Over half of crepuscular species have been recorded as being diel flexible, with approximately half of the diel flexible hypervolume being unique (Fig. 3c). Almost a fifth of cathemeral species are diel flexible with more than half of their hypervolume being unique, whilst 83% of the obligate cathemeral hypervolume was unique (Fig. 3d). Overall, 71% of the obligate diurnal hypervolume was unique (Fig. 3e).

## Discussion

Here, we show that, in mammals, species that have expanded their activity into the daytime show gradients of ecological strategies that differ from those adopted by night-active species. Our hypervolume analyses demonstrate that although nocturnal, cathemeral and diurnal diel niches maintain significant areas of unique trait space, there is also notable overlap in regions of trait space that may allow more species than currently realised to switch the timing of their activity in the face of anthropogenic change. Our findings yield the most comprehensive picture to date of how trait space is structured across the diel axis and reveal insights into the challenges and opportunities for mammals in a changing world.

The divergence in the axes of differentiation, and so differences in the ecological strategies between species using the nighttime and those using the daytime, is notable, and suggests that different abiotic and biotic selective forces play a greater role than unique histories in producing the observed patterns of trait diversification^21^. For night-active (nocturnal and crepuscular) species two dimensions of trait variation associated with PC1 and PC2 stand out. One dimension runs from large herbivorous species to small carnivorous species, while the second dimension runs from ground foraging species with large litter sizes to aerial species with small litter sizes. The position of these gradients is influenced by aerial foraging insectivorous bats, which make up almost a quarter of nocturnal and crepuscular species (753 and 34 species, respectively). To adapt to the high energetic costs of flight, insectivorous bats have lower productivity rates than other mammals, having small numbers of young and being long-lived for their size^22^. By contrast, PC1 in day-active (cathemeral and diurnal) species lies along a clear fast-slow continuum^23^. This axis reflects how quickly populations can recover from low levels as slow life-histories reduce the ability of populations to compensate for increased mortality^24^.

We also find that low-level foraging rodents and their allies associated with the first nocturnal, cathemeral and diurnal hotspots in trait space displayed marked differences in body mass and diet that are associated with geographical variation in habitat. In the tropics nocturnality dominates^25^, where the relatively reduced energetic demand of a warmer climate has driven the convergence on smaller body masses and on a lower energy but more predictable herbivorous diet. Conversely, cathemerality and diurnality dominate at the higher latitudes, where energetic demands associated with lower temperatures are greater^25,26^. The cathemeral hotspot that largely consists of shrews reflects a sporadic 24-h low-level foraging strategy on an energy-rich omnivorous or carnivorous diet, which has allowed them to maintain low body masses and produce relatively large litter sizes. Diurnal species in the first hotspot have more thermally efficient larger body masses, allowing the evolution of a broad range of dietary strategies. Considering that cathemeral species are approximately equally active at night as during the day the convergence in the axes of differentiation and the first hotspot with diurnal species is striking, suggesting that ecological and environmental conditions during the daytime drive major gradients of ecological strategies.

Phylogenetic constraints are known to play a role in determining time-partitioning strategies in mammals^25,27^, and we find that to be the case here. There was no marked variation in the strong phylogenetic conservatism between diel niches, demonstrating similar phylogenetic constraints across all diel strategies and that a species’ preferred activity time is influenced by their phylogenetic past. The number of families captured by hotspots gives an indication of the phylogenetic clustering of trait combinations^28^. The least number of families was captured by the two nocturnal hotspots (43%), revealing that relative to species in other diel niches, there is a low degree of clustering of families around similar trait combinations and that nocturnality persists across a broad range of evolutionary histories. Conversely, the two diurnal hotspots captured the greatest number of families (66%), demonstrating a relatively high degree of convergence in ecological strategies. The low number of families captured by the nocturnal compared to the diurnal hotspots is likely a result of the nocturnal bottleneck during the Mesozoic, which constrained taxonomic diversification^29^, but allowed time to evolve a greater diversity of ecological strategies. Strict diurnal species are thought to proliferate more rapidly than nocturnal species^30^, which supports increased speciation but decreased time to allow the diversification of traits. It is possible that following the demise of the dinosaurs, the repopulation of the diurnal niche with daylight-visual mammal, avian and reptilian predators and the resulting increase in competition and predation risk during the daytime ultimately deterred some species from switching from nocturnality to diurnality. The increased number of families captured by the three crepuscular (65% of families) compared to the single cathemeral (46% of families) hotspot is perhaps unsurprising, given that each hotspot occupies a distinct region of the ecological strategy surface and so multiple hotspots are more likely to capture species from a more diverse range of families.

We predict that the higher functional diversity, demonstrated by the larger hypervolume, and species richness of nocturnal mammals means that overall, the greater diversity of mammalian influence on ecosystem function and processes will occur at night. Over half of the nocturnal hypervolume (55%) is unique, which is explained in part by the large number of aerial foraging bats (*n* = 753). Although this strategy has been replicated in the crepuscular niche (*n* = 34) with species in the second nocturnal and crepuscular hotspot having evolved similar trait combinations, with the exception of a small number of bat populations this strategy has not been replicated in the daytime (e.g. ^18^). The fact that the unique functional trait space has not been maintained or replicated in day-active mammals suggests that these trait combinations may be less viable when faced with the different biotic and abiotic conditions during the daytime, particularly in the tropics where nocturnality dominates^25^. The small unique fraction of the non-nocturnal hypervolume (17%) demonstrates that most species that are not nocturnal retain the functional trait combinations for nocturnality, albeit with different major gradients in the axes of differentiation within diel niche ecological strategy surfaces. There was a high degree of overlap of the crepuscular with the nocturnal and diurnal hypervolumes. This reveals that in the functional traits analysed, crepuscular species are essentially both nocturnal and diurnal but have specialised to forage at dawn and dusk, possibly due to a combination of predator–prey relationships and to avoid competition with nocturnal and diurnal species^31^.

Dependent on the evolutionary history of the species, cathemerality is thought to have arisen for one of two reasons. First, being active during the nighttime and daytime might be a transition between strict nocturnal and strict diurnal activity, with this shift being driven by fluctuating environmental and ecological pressures^32^. This might be supported in our findings in two ways (1) the majority of the cathemeral hypervolume (82%) overlapped with the hypervolumes of other diel niches, and (2) the cathemeral hypervolume overlapped equally with the matched nocturnal and matched diurnal hypervolumes. Second, in either very small (e.g. shrews) or very large (e.g. elephants) species, cathemeral activity allows species to balance energetic and thermoregulatory demands by foraging sporadically across the daily cycle. This might be the case for species in the 18% of the cathemeral hypervolume that was uniquely cathemeral, which demonstrates how cathemerality is a successful strategy in its own right and one that has required the evolution of specific trait combinations. Over a quarter of diurnal trait space is unique suggesting that activity solely during the daytime has required the evolution of unique trait combinations that have not been maintained or replicated in the nighttime and this may particularly be the case at the higher latitudes where day-active strategies dominate^25^.

The major functional traits analysed here, although important for allowing inferences regarding the evolution of activity patterns, are not solely responsible for determining when a species is active. Specialised adaptations in some species may limit their ability to be active across diel niches. For example, enhanced visual acuity allows arboreal monkeys to exploit forest environments during the day^33^, visual adaptations coupled with group vigilance reduce predation risk in communal living diurnal species, such as Meerkats *Suricata suricatta*^34^, and the high energetic costs of flight^35^ coupled with reduced predation risk and competition^36^ keep most bat populations foraging at night. Missing data for a significant proportion of the 5104 species included in this analysis, meant that we were unable to explore the role of these traits in shaping activity patterns across mammals more broadly. However, the traits that we do include are known to summarise species response to environmental change, and as we know that the release from the environmental bottleneck was a major environmental change^1^ it is unsurprising that we see changes in these traits across diel niches. Less is known about the role of more subtle and complex species interactions, such as competition and predator–prey relationships, in shaping when species are active^37,38^. Where many species occur sympatrically, competing guilds can separate along a diel niche axis, for example gliding squirrels in tropical forests forage at night to avoid competition with diurnal tree squirrels^39^.

Despite only a fifth of mammals being recorded as active outside of their dominant diel niche, 86% of the hypervolume of these species overlapped with 44% of the hypervolume of apparently more obligate diel niches. The ability to switch diel niche is thus not confined to a unique corner of trait space but is distributed broadly and may therefore be more common than currently realised (with nocturnal activity by diurnal species also likely to be under-recorded). As nocturnality overlaps much of the trait space of other diel niches, generalised adaptations in mammals, such as in visual systems and eye morphology^40,41^ and energetics and resource use^26,42^ mean that many species may have the option to switch their activity patterns in the face of growing anthropogenic pressures. Switching to nocturnality, for example, appears to be a common, but not universal^43^, behavioural adaptation of day-active wildlife in response to largely diurnal human activity^15,44^. A similar shift to nocturnal activity may also compensate diurnal mammals for increased daytime temperatures as a result of anthropogenic climate change^15,45^. It is unclear how greater movement of mammals towards nighttime activity will impact communities and ecosystem processes and functions. However, when this is combined with the broad range of ecological impacts arising from the nighttime’s own rapidly increasing—and uniquely nocturnal—pressure of artificial light^46^, there may be profound consequences for species already active at night.

Our analysis also revealed that over half of the hypervolume (56%) of species with obligate diel niches was unique and therefore the functional traits of these species may not allow them to switch their activity patterns. The majority of these species are obligately nocturnal, which approximately matches the size of the unique portion of the nocturnal trait hypervolume in the first hypervolume analysis. This will be important for species’ adaptation and ultimately survival in the face of anthropogenic pressures, with obligate nocturnal and obligate diurnal species having been shown to be more than twice as likely to respond to climate change (e.g. through expiration or range shift) than species with flexible activity patterns^47^. Nocturnal species with larger body sizes have also been found to exhibit positive habitat gains, emphasizing the importance of considering multiple traits for understanding climate change impacts^47^.

Over half of crepuscular species were found to be diel flexible, being able to switch the timing of their dominant activity under fluctuating environmental and ecological conditions. Approximately half of the diel flexible crepuscular hypervolume was unique, indicating that these species show different functional trait combinations than other crepuscular species and may be more representative of nocturnal or diurnal species. Almost a fifth of cathemeral species are diel flexible, with more than half of their hypervolume being unique. In cathemeral species, being diel flexible generally equates to the ability to be cathemeral in one season and nocturnal or diurnal in a different season. The ability to switch activity patterns in this way may require certain trait combinations that are not realised in most cathemeral species. Conversely, the majority of the obligate cathemeral hypervolume was unique, demonstrating that from a functional trait perspective, cathemerality as a transition between strict nocturnal and strict diurnal activity may occur over an evolutionary as opposed to an ecological timescale.

Almost three quarters of the obligate diurnal hypervolume was unique, suggesting that the greater proportion of mammals that moved into the daytime do not possess trait combinations that allow them to switch back into the nighttime in the face of anthropogenic pressures. Obligate diurnal mammals are particularly prone to climate warming, with diurnal habitat space predicted to be lost under all warming scenarios^26^. This effect is compounded by asymmetric warming across the diel cycle over much of the land’s surface^48^. In regions where the daytime has warmed more, there has been a trend of greater overall warming and a drying of the climate^48^ making diurnal species increasingly vulnerable to heat exhaustion and water loss^49^. Conversely, greater nighttime warming has been found to be twice as common as daytime warming^48^. In some regions, such as at higher latitudes where cold nighttime temperatures limit activity, this may allow nocturnal mammals to gain habitable space unless they have low body mass or high thermal conductance^26^. In other regions, such as low elevation tropical latitudes, it erodes the ability of the nighttime to act as a ‘thermal refuge’ where species can recover from daytime heat stress^16^. The ability to change activity patterns in response to less favourable daytime or nightime environments may help species adaptation to climate change and determine their future distributions and survival.

In the same way that habitat specialists are more likely to be threatened by habitat loss, or large-bodied species are more likely to be threatened by overexploitation^24^, when a species is active may determine not only its risk of extinction from anthropogenic activities but also our awareness of this risk^25^. It is unclear how anthropogenic activities impact species behaviour and competitive interactions via altered activity patterns and diel niche partitioning and understanding these impacts may shed light on the underlying mechanisms driving changes to species distributions and population size in human-modified landscapes. This will be important, for example, because the switching in the diel niche of an apex predator can not only influence ecosystems by decreasing prey abundance and changing prey behaviour^50^, but can also cause a behavioural mediated cascade whereby mesopredators switch to an opposing diel niche as they may perceive humans to be less dangerous than apex predators^43,51^. Untangling how these intrinsic and extrinsic interactions are changing across the diel axis is therefore an important component for designing appropriate management strategies to facilitate human-wildlife coexistence and support biodiversity in impacted landscapes^52^.

There has been much interest in applying trait-based research to guide conservation policies and actions to maximise the functional diversity that is conserved in the face of anthropogenic pressures^11,53–55^. We show that much of the unique functional diversity in mammals occurs in those species that are active at night and nocturnal species therefore have potentially irreplaceable ecological roles which, if lost, could undermine the integrity of ecological processes and functions^53,56^. Despite around 30% of all vertebrates and more than 60% of all invertebrates globally being nocturnal^57^, relatively little is known about their functional diversity and how it differs from day-active species^38^. Challenges in conducting scientific research at night mean that much of what we know about ecology comes from studies on daytime-active species and this may complicate the surveying, monitoring and ultimately the conservation of night active species to protect the ecosystem processes and functions they help to support. Diel variation in functional diversity highlights how the potential ecological costs of species loss vary across the diel cycle, and therefore provides a complementary perspective to the ecological distinctiveness framework^53^. To best maintain ecosystem viability in the face of anthropogenic change it will be necessary to expand understanding not only of what ecological processes occur but also when they occur. In this way, we might also understand how the ability of species to be flexible in their activity patterns might act to protect or erode biodiversity and ecosystem functioning into the future.

## Methods

### Summary

Following the Handbook of Mammals of the World (Volumes 1–3 & 5–9^58^), we assembled a database of activity patterns for 5104 extant mammals representing 25 of the 29 extant orders and 133 of the 148 extant families. We excluded sea mammals (including two species of marine otter *Enhydra lutris* and *Lontra felina*) and fossorial species because these are likely to be reliant on different light cues than above surface terrestrial species. We assigned each species to one of four activity patterns: (1) nocturnal—active only at night; (2) crepuscular—active only during twilight (around sunrise and sunset); (3) cathemeral—active throughout the day and night, interspersed with rest periods; (4) diurnal—active only during the day (Supplementary Methods 1). Flexibility in the timing of when a species is active demonstrates an ability to adapt to environmental change and anthropogenic pressures^6,16^, and because it is active across a broader diel niche it has the potential for a greater contribution to ecosystem processes and functions. We therefore assigned each species as being diel flexible if there was evidence of activity outside of its dominant activity pattern, otherwise it was recorded as diel obligate (Supplementary Methods 1).

We collated information on a further five major traits that summarise a species’ form, function and ecological strategy: body mass (mean adult body mass in grams), litter size (mean number of offspring per reproductive effort), diet (a continuous synthetic trait generated from ten diet categories, see below), foraging strata (treated as ordinal; ground, scansorial, arboreal, aerial), and habitat breadth (number of IUCN habitats listed as suitable; Supplementary Methods 1). These traits dictate both a species’ influence on ecological and biogeochemical processes and how they respond to change. All data processing and analyses were performed in R software for statistical computing v3.5.2^59^. Citations for R functions, packages and package versions used can be found in Supplementary Table 4.

Based on semi-quantitative records of ten different diet categories, we calculated a continuous measure of a species’ diet. We first calculated Gower Distances between species based on the diet data using the gowdis() function in the FD package (Supplementary Table 4; Supplementary Methods 1), before performing a principal component analysis (PCoA) on the Gower distances using the dudi-pco() function in the ade4 package (Supplementary Table 4). The first principal component axis captured 40.7% of variation and, following^12^, only these values were used to serve as synthetic trait values (i.e. new trait values based on the relative importance of diet categories in the initial dataset; but see Supplementary Methods 1 for details on analyses also including the second principal component axis that captured 18.7% of variation). Diet was predominantly loaded positively on invertebrates (PCoA loading = 0.091) and vertebrates (0.023) and negatively on plant material and seeds (−0.073; Supplementary Fig. 1), thus representing a gradient from invertivore to herbivore, reflecting previous diet ordinations for mammals^12,60^.

Body mass, litter size, diet and habitat breadth underwent a stringent selection procedure by Cooke et al.^53^ and were found to be highly suitable for analyses in functional trait space. Cooke et al.^53^ included volancy (the ability to fly) instead of foraging strata because foraging strata had >50% missing values and their analyses examined the roles of birds and mammals. Here we include foraging strata because (1) we were able to achieve <4% missing values, and (2) it provides a deeper understanding of where the ecological impacts of a mammal’s foraging will occur. High multicollinearity between traits can potentially obscure the functional value of the correlated traits^8^. We tested for multicollinearity between our five traits by regressing all traits in a multinomial model against activity pattern as the response and assessing Variance Inflation Factors (VIF; vif function in the car package (Supplementary Table 4). All traits had a VIF value of <4.

Trait data were transformed where this improved normality: log_10_ for body mass and litter size, and square root for habitat breadth; all traits were standardised to zero mean and unit variance (z-transformation) as recommended for trait analyses^7,61^ and hypervolume calculations^62^.

### Trait imputation

Trait data were not available for all species but, overall, less than 5% of trait values were missing (Supplementary Table 6). Excluding species with incomplete data (data-deletion approach) reduces sample sizes (and consequently the statistical power of the analysis), may introduce bias^63,64^, and would restrict the dimensionality of the analysis^62^. To achieve complete species-trait coverage, we imputed missing data for activity patterns (3%), body mass (1%), litter size (24%), diet (2%), foraging strata (1%) and habitat breath (<1%; see Supplementary Methods 2 for a full methodology of data imputation for missing species, and Supplementary Table 6 for a summary of trait coverage). Phylogenetic data can improve the estimation of missing trait values in the imputation process, because closely related species tend to be more similar to each other and many traits display high degrees of phylogenetic signal^65^. To generate imputed values, we used the mice function from the Multivariate Imputation with Chained Equations (MICE) package (Supplementary Table 4) based on the ecological (the transformed traits) and phylogenetic (the first ten phylogenetic eigenvectors extracted from trees obtained from PHYLACINE 1.2 database^3,63^) relationships between species. MICE has been shown to have greater accuracy, improved sample size and smaller error and bias than single imputation methods and the data-deletion approach^63,64^. Following Cooke et al.^53^, we extracted 25 inputted datasets and repeated the imputations 100 times per dataset (i.e. 2500 imputed values). We then calculated the mean missing value across datasets. To test the reliability of our imputed data, we also ran the analyses excluding species with missing data (i.e., following the data-deletion approach, *n* = 3794, see Sensitivity Tests below). We provide the datasets without and with mean imputed data (Supplementary Data 2) and all 25 datasets with imputed data (Supplementary Data 3).

The strongest correlations across traits were between body mass and diet (Pearson’s correlation (*r*) = −0.55), and foraging strata and litter size (*r* = 0.59), whereas the weakest correlations were between foraging strata and habitat (*r* = −0.03) and litter size and diet (*r* = 0.001).

### Phylogenetic analysis

A phylogenetic supertree was available from the PHYLACINE 1.2 database for all 5104 mammals^3^. Phylogenetic data are not available for all species, the PHYLACINE 1.2 database employs a hierarchical Bayesian approach to provide a posterior distribution of 1000 trees, which is intended to recover uncertainties in topology and branch length of missing species (PHYLACINE 1.2 metadata). To avoid circular reasoning, we omitted species from the phylogenetic analysis for which we had imputed activity pattern (*n* = 164). We randomly selected 30 trees and using the fitDiscrete() function in the geiger package (Supplementary Table 4) we calculated Pagel’s *λ* for activity patterns for all retained species (*n* = 4940) for each tree in turn, before giving the mean and standard deviation. We then calculated Pagel’s *λ* for each diel niche. For the nocturnal niche, we assigned each retained species as nocturnal or non-nocturnal, before calculating Pagel’s *λ* for the same 30 randomly selected trees as above. This approach was then repeated for the remaining diel niches.

### Ecological strategies between diel niches

For each activity pattern in turn we built a two-dimensional ecological strategy surface from the transformed and standardised traits via PCA, using the princomp() function in the vegan package (Supplementary Table 4). The ordination of species across this surface represents a two-dimensional continuum, integrating ecological strategies within each of the five trait dimensions.

We used multivariate kernel density estimation to calculate the occurrence probability of given combinations of trait values (probability contours) across the ecological strategy surface^28^, via the kde() function in the ks package (Supplementary Table 4). We extracted contours at the 0.5, 0.95 and 0.99 quantiles of the probability distribution, thus highlighting the regions of highest and lowest trait occurrence probability (Fig. 1). Because the results depend on the choice of the bandwidth used for the smoothing kernel, we used unconstrained bandwidth selectors that were the sum of the asymptotic mean squared error pilot bandwidth selector^66^, through the Hpi() function in the ks package (Supplementary Table 4).

### Hypervolume estimation

To evaluate n-dimensional functional trait space of mammals occupying different diel niches, we employed hypervolume estimation. The hypervolume approach is a way to measure accurately the volume of a high-dimensional shape without assuming a parametric probability distribution^62,67^ and can capture holes, disjunctions or other complex geometrical features, and thus hypervolumes model multidimensional spaces better than linear and continuous dimensions, such as convex hulls^68^.

Broadly, following the methodology from Cooke et al.^12^, we built a hypervolume for each diel niche using the one-class Support Vector Machine (SVM) estimation method^68^. SVM provides a smooth fit around the data that is insensitive to outliers, yields a binary boundary classification (in or out), is invariant to rotational transformation (i.e., correlations between axes) and is computationally viable in large datasets and high-dimensional hyperspaces^67^. We used the SVM method as extreme values in the observed data were considered to represent the true boundaries of our data^67^. We calculated the observed hypervolume based on the transformed and standardised traits using the hypervolume_svm() function in the hypervolume package (Supplementary Table 4). Conversion to unitless coordinates (here z-transformation) is required so that volumes or overlaps can be defined^62,67^. The units of the hypervolumes are reported as the standard deviations of centred and scaled transformed trait values, raised to the power of the number of dimensions (SD^number of dimensions^).

To enable comparison between hypervolumes of each diel niche and all species of the other diel niches, for each diel niche hypervolume, we then built a second hypervolume of all species excluding the diel niche of interest. We first recorded the volume of each hypervolume, before assessing pairwise overlap among the ten paired hypervolumes using the hypervolume_overlap_statistics() function in the hypervolume package (four pairs of each diel niche hypervolume compared with hypervolumes of all other species, and six pairs of each diel niche hypervolume with each of the other diel niche hypervolumes; Supplementary Table 4). This function calculates the unique volume fraction of each hypervolume. We assessed pairwise overlap between (1) each diel niche and all other species (four paired hypervolumes in total), and (2) each diel niche with matched sample sizes of each of the other diel niches in turn (six paired hypervolumes in total).

Comparative statistics can be influenced by sample size^20^. Therefore, as the number of species occupying each diel niche varies strongly across niches, for the comparison of individual diel niche hypervolumes, we selected a random sample of species from the larger of each paired hypervolume to match the number of species in the smaller hypervolume. To ensure that our results were not biased by the species selected in the random sample, we repeated each comparative analysis on 100 random subsets from the larger hypervolume, before calculating the mean value for each statistic. We were interested in the total volume of trait space occupied by each diel niche relative to the total trait space occupied by all other species, and so for the first paired analysis, we did not match sample sizes.

### Diel flexibility in trait space

We examined whether mammals that displayed diel flexibility in when they are active occupied a distinct region of trait space, or whether flexibility in activity patterns occurred across a broad range of functional trait combinations. We first built hypervolumes for all diel flexible species (*n* = 919), and all diel obligate species (*n* = 4185), before carrying out pairwise comparative analysis to calculate the unique volume fraction in the diel flexible and diel obligate hypervolumes. To unpick which trait combinations allow flexibility in the timing of activity within each diel niche, we constructed a hypervolume of diel flexible species and a hypervolume of diel obligate species for each diel niche and recorded the volume. We then calculated the unique volume fraction of each hypervolume in each pair.

### Sensitivity tests

Overall, our results and conclusions were qualitatively similar (1) with and without imputed data (Supplementary Tables 1b, 2b, 5; Supplementary Figs. 3–5), (2) when including the first or the first and second principal components from the diet PCoA (Supplementary Fig. 2), and (3) were robust with respect to the identity of the traits (Supplementary Tables 7 and 8), and (4) when including activity pattern as a sixth trait in the ecological strategy surface. Further, information on these analyses is provided in Supplementary Methods 3.

### Reporting summary

Further information on research design is available in the Nature Research Reporting Summary linked to this article.

## Supplementary information

Supplementary Information

Reporting Summary

Description of Additional Supplementary Files

Supplementary Data 1

Supplementary Data 2

Supplementary Data 3

## Data Availability

The trait data were extracted principally from the Handbook of the Mammals of the World (Volumes 1–3 & 5–9^58^), PHYLACINE 1.2^3^, Cooke et al.^9^ and EltonTraits 1.0^69^. The following three datasets are available on figshare (10.6084/m9.figshare.13623014): Supplementary Data 1, Taxonomic composition of functional hotspots in Fig. 1; Supplementary Data 2, Trait data containing both missing values and imputed data, with data sources; Supplementary Data 3, 25 datasets containing imputed data. Phylogenetic data was downloaded from PHYLACINE 1.2^3^, and are available on the Dryad Digital Data Repository (10.5061/dryad.bp26v20). Fig. 1 was produced by 2-dimensional ordination of raw trait data. Figs. 2–3 are two-dimensional representations of five-dimensional trait space generated from raw trait data.

## Code availability

Code for the production of Fig. 1 was adapted from Díaz et al.^28^ (ftp://pbil.univ-lyon1.fr/pub/datasets/dray/Diaz_Nature/). Code used in the analysis was based on cited packages in the R Statistical Environment^59^ (Supplementary Table 4).
