# Supplementary material for: Diel niche variation in mammals associated with expanded trait space
Source: Nat Commun. 2021 Mar 19;12:1753. doi: 10.1038/s41467-021-22023-4 (PMC7979707; doi:10.1038/s41467-021-22023-4)
Supplement: Supplementary file 1 — Supplementary Information [file 41467_2021_22023_MOESM1_ESM.pdf]

Diel niche variation in mammals associated with expanded trait space

Cox DTC*, Gardner AS, Gaston KJ

Environment and Sustainability Institute, University of Exeter, Penryn, Cornwall, TR10 9FE, U.K.

*Corresponding author: Daniel TC Cox: d.t.c.cox@exeter.ac.uk; Phone: +44 (0) 7800556070

Supplementary Methods 1: Assembly of trait data

Supplementary Methods 2: Imputation of missing species

Supplementary Methods 3: Sensitivity tests

Supplementary Table 1: Ecological strategy surface 1: Trait variance

Supplementary Table 2: Ecological strategy surface 2: Trait loading

Supplementary Table 3: Hypervolume comparative statistics

Supplementary Table 4: R Functions, packages and package versions used in the analysis

Supplementary Table 5: Hypervolume comparative statistics under the data deletion approach

Supplementary Table 6: Traits selected, data type and percentage coverage

Supplementary Table 7: Tests of sensitivity to trait selection: Diel niche hypervolumes

Supplementary Table 8: Tests of sensitivity to trait selection 2: Diel flexibility hypervolumes

Supplementary Table 9: Hotspot analysis of the ecological strategy surface of all six functional traits

Supplementary Fig. 1: Principal Coordinates Analysis (PCoA) of diet categories

Supplementary Fig. 2: Ecological strategy surfaces for each diel niche including the first two synthetic diet traits

Supplementary Fig. 3: Ecological strategy surfaces for each diel niche under the data deletion approach

Supplementary Fig. 4: Two-dimensional representation of functional trait space under the data deletion approach

Supplementary Fig. 5: Flexible and obligate hypervolumes under the data deletion approach

Supplementary Fig. 6: Ecological strategy surface of mammals for six functional traits

Supplementary Methods 1

Activity patterns

We collated activity records for 5,104 mammal species, representing 25 of the 29 extant orders and 133 of the 148 extant families from the Handbook of the Mammals of the World volumes 1-3 and 5-9[1–8]. The Handbook of the Mammals of the World is the most complete resource available for mammalian activity patterns, covering all mammal species described at the time of writing. For taxonomy, we followed the Phylogenetic Atlas of Mammal Macroecology (PHYLACINE 1.2[9]). This taxonomically integrated platform contains phylogenies, range maps, trait data, and threat status for all 5,831 known mammal species that have lived since the last interglacial. Following the IUCN Red List for threatened species we excluded extinct mammals (n = 353) unless they were recently extinct and represented in the Handbook of Mammals of the World (n = 5). We resolved conflicts where sources disagreed on taxonomy as follows: (1) where there was conflict between the Handbook of Mammals of the World and the PHYLACINE 1.2 dataset in genus and species names, we followed PHYLACINE 1.2; (2) for species recorded as full species by PHYLACINE 1.2 but were not full species in the Handbook of Mammals of the World (e.g. subspecies or races), we followed PHYLACINE 1.2 and recorded the activity patterns of the species that the subspecies was classified under in the Handbook of Mammals of the World; (3) species which were present in the Handbook of Mammals of the World but not in PHYLACINE 1.2 were excluded from the analysis (n = 832). We excluded mammals that live primarily in the marine environment (n = 127; found in the Handbook of Mammals of the World volume IV[10] (including two species of marine otter *Enhydra lutris* and *Lontra felina*) and species described as highly or fully fossorial (n = 247), because these species are likely to be reliant on different light cues than above surface terrestrial species.

Based on their known predominant activity pattern, we assigned each species as one of the following: (1) nocturnal – active only at night; (2) crepuscular – active only during twilight at

around sunrise and/or sunset; (3) cathemeral – active throughout the day and night, interspersed with rest periods; (4) diurnal – active only during the day. Those species that varied the timing of their activity across the seasons were assigned patterns based on their predominant pattern, or if that information was not available, their activity patterns during their breeding season. All screening was carried out by two researchers. A subset of 70 species were screened by both researchers to ensure consistency in recording. Data on activity patterns was successfully obtained for 4,301 species. For the 639 species for which no information was available in the Handbook of Mammals of the World, activity patterns were identified through an extensive literature search of books, peer-reviewed literature and their supplementary material and grey literature. Activity pattern for the remaining 165 species were estimated through data imputation (see below).

Flexibility in when a species is active demonstrates an ability to adapt to environmental change and anthropogenic pressures[11], and because it is active across a broader diel niche suggests the potential for greater contribution to ecosystem processes and functions. Therefore, if a species had been recorded in the Handbook of Mammals of the World or in the wider literature (e.g.[11–13]) as being active outside of its dominant activity period, we recorded the species as having flexible activity patterns, otherwise they were recorded as diel obligate. For example, where a species was described as ‘predominantly nocturnal’ we assigned the species as nocturnal and flexible, whereas a species described as nocturnal or strictly nocturnal were recorded as nocturnal. Records of crepuscular activity (dusk or dawn), when in conjunction with nocturnal or diurnal activity, were recorded as nocturnal or diurnal respectively and flexible. Species that varied their activity patterns across the seasons were also recorded as being flexible, for example cathemeral species that become diurnal during winter. Species that have only been recorded as active outside of their dominant diel niche in laboratory conditions were recorded as diel obligate, because this may not happen in the wild and so the ecological impact of occupying a different diel niche may not be realised.

Functional traits

The contribution of species to ecosystem processes, such as pollination, predation, herbivory and seed dispersal, depends on biological traits related to the spatiotemporal distribution of resource capture, utilisation and release[14–19]. Here, we selected five major traits that summarise a species' form, function and ecological strategy, namely body mass, litter size, diet, foraging strata and habitat breadth. These traits dictate both a species' influence on ecological and biogeochemical processes and how they respond to change.

Body mass (numeric) – Body mass reflects the type and amount of resources that a species consumes and releases[16], and underlies many of their physiological, ecological and evolutionary processes, and therefore their contribution to various functions[21–26]. Body mass data were sourced from PHYLACINE 1.2 and we included only those body masses that were reported or were estimated from morphological correlates based on a relative that a species was said closely to resemble in size[9]. We excluded body mass for 173 species from PHYLACINE 1.2 that were estimated by phylogenetic imputation, instead we included species mean body mass from the Handbook of Mammals of the World and from literature searches (n = 105). The mean body masses for the remaining 68 species were estimated through data imputation.

Litter size (numeric): Litter size relates to a species' reproductive strategy and output (fecundity) and therefore its contribution to trophic processes, such as herbivory (routing basal energy into food-webs via many offspring). A diversity of reproductive strategies across an ecosystem may be important for coping with change[9]. Data for litter size were obtained from Cooke et al.[20], who sourced data from the Amniote and PanTHERIA databases, where litter sizes were present in both databases (46%) they calculated the mean across databases (n = 3,136). Litter size for a further 733 species was sourced from the Handbook of the Mammals of the World, whilst data for the remaining 1,235 species were estimated through data imputation.

Diet (numerical): The impact of mammals on ecosystem function is related primarily to what they eat and how they procure their food[16]. Following Cooke et al.[20] and Pineda-Munzo and Alroy[27] we calculated a continuous measure of a species' diet. Raw diet information was available from the EltonTraits 1.0 database as semi-quantitative records (percentage use of ten different dietary categories) for 4,782 species[28]. For the 322 species not included in the EltonTraits 1.0 database, following the EltonTraits methodology[28], we quantified diet from the Handbook of Mammals of the World and from the literature (n = 206). No diet data were available for 116 species, for these we estimated diet based on diet categories phylogenetically imputed by PHYLACINE 1.2. Due to their reliability for accurately imputing missing data, PHYACINE 1.2 recorded diet in three categories (vertebrate prey, invertebrate prey and plants[29]). By interpolation at genus and family level, we then disaggregated the coarse vertebrate prey and plant categories into the corresponding five and four categories, respectively, employed by Elton Traits 1.0. To convert diet into a continuous measure, we first calculated Gower Distances between species based on the diet data, using the gowdis() function in the 'FD' package[30]. We then performed a Principal Coordinates Analysis (PCoA) on the Gower distances using the dudi-pco() function in the 'ade4' package[31]. PCoA rotates the matrix of Gower distances to summarise inter-species change in positions of the species relative to each other but changes the coordinate system. Trait space and hypervolume analyses assume that all axes contribute equally to distances and volumes. The first principal component from the mammal diet PCoA sufficiently captured variation across species[1], and so we only used these values to serve as synthetic trait values (i.e. new trait values based on the relative importance of diet categories in the initial dataset) and are referred to as diet. Diet explained 41% of the variation across the diet categories and was predominantly loaded positively on invertebrates (PCoA loading = 0.119) and vertebrates (0.023), and negatively on plant material and seeds (-0.098), thus representing a gradient from invertivore to herbivore (Supplementary Fig. 1), reflecting previous diet ordination for mammals[20,27].

Foraging strata (integer) – The strata at which a species forages shape the spatial dimensionality of resource use, with species consistently evolving distinct sets of morphological traits associated with foraging strata[32]. Data were obtained from EltonTraits 1.0 (n = 4,782[28]). Each species was assigned one of four foraging stratum categories, before transforming to an integer where: Ground level (including aquatic foraging) = 1; Scansorial = 2; Arboreal = 3; Aerial = 4. Following the protocol in EltonTraits 1.0, we obtained data for a further 278 species from the Handbook of Mammals of the World and literature searches. Foraging strata for the remaining 44 species were estimated through data imputation.

Habitat breadth (numeric): Habitat use strongly influences the spatial distribution and extent of resource intake and release by species, and thus the functional influence of a species across habitat types[16]. Data for habitat breadth were extracted from the IUCN Habitats Classification Scheme (http://www.iucnredlist.org/technical-documents/classification-schemes/habitats-classification-scheme-ver3; n = 5,070). Habitat breadth was then quantified as the number of habitats listed for each species. Following the IUCN Habitats Classification Scheme protocol, data for missing species were obtained from the Handbook of Mammals of the World and literature searches (n = 31). Those for the remaining three species were estimated through data imputation.

Supplementary Methods 2

To impute missing trait data and so avoid potential pitfalls and biases of reduced sample sizes from excluding species with missing trait data (e.g.[33,34]), we implemented Multivariate Imputation with Chained Equations (MICE). MICE has been found to have smaller error and bias compared to other multiple imputation approaches[33,35]. The pattern of missing data throughout our dataset lends itself to multiple imputation, with almost three quarters of species having complete datasets (c.74%). Only 6% of mammals suffered from missing data in more than one trait, and only 3% had missing

data in more than two traits. Phylogenetic data can improve the estimation of missing trait values in the imputation process[36–40], because closely related species tend to be more similar to each other[41] and many traits display high degrees of phylogenetic signal[42]. Phylogenetic information was therefore summarised by eigenvectors extracted from a principal component analysis, representing the variation in the phylogenetic distances among species[43,44].

A phylogenetic supertree was available from the PHYLACINE 1.2 database for all 5,104 mammals[9]. Because phylogenetic data are not available for all species, the PHYLACINE 1.2 database provides a posterior distribution of 1,000 trees, which is intended to recover uncertainties in topology and branch length of missing species (see PHYLACINE 1.2 metadata). Due to heavy computing requirements, we randomly selected 200 trees, before decomposing each into a set of orthogonal phylogenetic eigenvectors using the *PVRdecomp* function (‘PVR’ package[45]). We then selected the first 10 eigenvectors, because this number has been shown to minimise error in the imputations[46] and calculated the mean for each eigenvector and species across all 200 trees. Note, that these eigenvectors are more representative of divergences closer to the root of the phylogeny so they do not include fine-scale differences among species[42]. The first 10 eigenvectors represented 59% of the variation in the phylogenetic distances.

To generate imputed values, we used the *mice* function (‘mice’ package[45]). We specified the method as: predictive mean matching for continuous traits, which preserves non-linear relationships[45] often present in trait datasets[46]; logistic regression for binary traits; and polytomous regression for the categorical traits[45]. Variables included in the *mice* function were the transformed traits and the first 10 phylogenetic eigenvectors. Following Cooke et al.[1], we extracted 25 imputed datasets and repeated the imputations 100 times per dataset (i.e. 2,500 imputed values). To ensure inputted values most closely resembled real values, we calculated the mean value across the imputed datasets.

Supplementary Methods 3

To test the robustness of our results we took the following approaches: (1) Following Cooke et al.[1] we used only the first principal component from the diet PCoA for our main analyses, so that each trait dimension had equal weight. However, for comparison we provide the ecological strategy surface for each diel niche including the first and second principal components from the diet PCoA (Supplementary Fig. 2). (2) Under the data deletion approach (n = 3,793), we repeated the ecological strategy surfaces (Supplementary Table 1b and 2b; Supplementary Fig. 3) and the comparative estimation for paired diel niche hypervolumes (Supplementary Table 5a-c; Supplementary Fig. 4) and diel flexible vs diel obligate hypervolumes (Supplementary Table 5d; Supplementary Fig. 5). (3) We performed trait exclusion tests, to test whether the results shown in Fig. 2 and Fig. 3 were robust with respect to the traits selected. These tests excluded one individual trait at a time, before generating hypervolumes and carrying out comparative analyses between hypervolume pairs. We excluded individual traits from comparisons of (a) each diel niche hypervolume with the hypervolume of all species from other diel niches (Supplementary Table 7), and (b) the hypervolumes of diel flexible species with the hypervolumes of diel obligate species (Supplementary Table 8). (4) We tested whether diel niche differentiates species in trait space more than other traits. We included activity pattern as a sixth trait in an ecological strategy surface, thus establishing if the general patterns between diel niches demonstrated by the main analysis hold true. We first calculated Gower Distances between species based on transformed traits, including activity pattern as a categorical variable (gowdis() function in the ‘FD’ package[30]). We then performed a principal component analysis (PCoA) on the Gower Distances using the dudi-pco() function in the ‘ade4’ package[31], before carrying out multivariate kernel density estimation as in the main analysis (Supplementary Fig. 6). We repeated the hotspot analysis to determine areas of particularly dense species occupation across diel niches (Supplementary Table 9).

Overall our results were qualitatively similar (1) with both synthetic diet trait values (compare Fig. 2 with Supplementary Fig. 2), (2) with and without imputed data (compare Fig. 1-3 with Supplementary Fig. 3-5), and (3) when excluding a fifth trait (compare Table 1 with Supplementary Table 7, and Table 4b with Supplementary Table 8). (4) Ordination of six traits revealed distinct separation in trait space of nocturnal, cathemeral and diurnal species (Supplementary Fig. 6). The ecological strategy surface shows similar axes of differentiation to nocturnal and crepuscular species, driven by high speciation but low trait diversification of the order Chiroptera (hotspots 3 & 4). There was distinct separation on the ecological strategy surface between the centroids of each diel niche. The nocturnal centroid was located on higher positive values on PC1 than other centroids and was positioned along a gradient of ground foraging species with large litter sizes, to small litter sizes in aerial foraging insectivorous bats. In response to extensive overlap in trait space with other diel niches, albeit at low densities, the crepuscular centroid was centred between the remaining three diel niche centroids. The cathemeral centroid was located on positive values of PC2 driven by the large litter sizes and small body masses of shrews (Hotspot 10). Diurnal centroid was located on lower values of PC1 and PC2, in response to the arboreal primates (hotspots 6 & 7), large herbivores, and rodents (hotspot 9). Hotspots capture areas of dense species occupation and incorporate 50% of all species in each diel niche. There were ten hotspots that captured 65% of the same species captured in the main analysis (1,663 species). Nocturnal species dominated five hotspots, cathemeral species two hotspots and diurnal species three hotspots (Supplementary Table 9; Supplementary Fig. 6).

Supplementary Table 1: Ecological strategy surfaces 1. For each diel niche the proportion of variance accounted for by each of the functional traits, and the variance explained by the first two principle components (PC) of the PCoA analysis, under (a) data imputation (n = 5,104), and (b) the data deletion approach (n = 3,793).

	Nocturnal	Crepuscular	Cathemeral	Diurnal
(a) Data imputation				
Body mass	24.9	24.2	24.1	25.1
Litter size	24.1	25.4	20.8	24.6
Diet	18.4	21.7	16.8	7.4
Foraging strata	24.9	23.0	19.4	21.5
Habitat breadth	7.7	5.8	18.8	21.0
PC 1 (%)	41.4	52.9	48.1	39.7
PC 2 (%)	25.2	22.7	24.0	24.8
(b) Data deletion				
Body mass	24.5	24.1	24.4	25.6
Litter size	21.9	25.5	20.9	24.2
Diet	17.8	21.8	16.5	7.8
Foraging strata	24.9	22.8	19.0	21.1
Habitat breadth	10.8	5.8	19.2	21.3
PC 1 (%)	42.5	52.8	46.7	38.6
PC 2 (%)	23.4	22.8	24.3	25.3

Supplementary Table 2: Ecological strategy surfaces 2. For each diel niche the loading on each trait for the first and second axis of differentiation under (a) data imputation (n = 5,104), and (b) data deletion (n = 3,793).

	Nocturnal		Crepuscular		Cathemeral		Diurnal	
(a) *Data imputation*								
	PC1	*PC2*	*PC1 PC2*	*PC2*	*PC1*	*PC2*	*PC1*	*PC2*
Body mass	-0.41	-0.62	-0.53	-0.40	-0.60	-	-0.57	-0.36
Litter size	-0.46	0.54	-0.22	0.86	0.53	-0.24	0.63	-
Diet	0.50	0.28	0.55	0.11	0.50	-	0.34	-
Foraging strata	0.61	-0.23	0.55	-0.25	-0.20	-0.70	-0.40	0.54
Habitat breadth	-	-0.45	-0.27	-0.16	-0.27	-0.65	-0.26	-0.75
(b) *Data deletion*								
	PC1	*PC1*	*PC1 PC2*	*PC2*	*PC1*	*PC2*	*PC1*	*PC2*
Body mass	-0.44	-0.59	-0.53	-0.40	-0.60	-0.11	-0.57	-0.38
Litter size	-0.43	0.53	-0.20	0.87	0.54	-0.24	0.63	-
Diet	0.51	0.17	0.55	0.10	0.50	-	0.35	-
Foraging strata	0.60	-0.21	0.55	-0.24	-0.21	0.69	-0.39	0.55
Habitat breadth	-	-0.55	-0.27	-0.14	-0.24	-0.67	-	-0.73

Supplementary Table 3: Comparative statistics of (a) hypervolumes of all species in each diel niche with all species in a different diel niche under the data imputation approach: Nocturnal, n = 3,580; Crepuscular, n = 126; Cathemeral, n = 467; Diurnal, n = 931 (Data used to create Fig. 2), and (b) hypervolumes of flexible species with hypervolumes of obligate species (Data used to create Fig. 3). We give the volume and the unique fraction of each hypervolume.

**Hypervolume 1 (H1)**	**Hypervolume 2 (H2)**	**Volume H1** (SD^5)	**Volume H2** (SD^5)	**Unique fraction H1**	**Unique fraction H2**
(a) Fig. 2: Activity patterns with all species					
Nocturnal	Crepuscular	375	43	0.89	0.01
Nocturnal	Cathemeral	375	96	0.79	0.19
Nocturnal	Diurnal	375	226	0.57	0.29
Crepuscular	Cathemeral	43	95	0.32	0.70
Crepuscular	Diurnal	43	222	0.08	0.83
Cathemeral	Diurnal	96	222	0.29	0.68
(b) Fig. 3: Flexible and obligate					
All flexible all	All obligate species	182	357	0.14	0.56
Flexible nocturnal	Obligate nocturnal	179	360	0.19	0.59
Flexible crepuscular	Obligate crepuscular	20	22	0.50	0.54
Flexible cathemeral	Obligate cathemeral	26	67	0.57	0.83
Flexible diurnal	Obligate diurnal	76	200	0.22	0.71

Supplementary Table 4: R Functions, packages and package versions used in the analysis and cited in the main text.

Function	Package; version	Citation
gowdis()	FD; 1.0-12	Laliberté, E., Legendre, P. & Shipley, B. FD: measuring functional diversity from multiple traits, and other tools for functional ecology. (2014).
dudi-pco()	ade4; 5.3	Dray, S. & Dufour, A.-B. The 'ade4' Package: implementing the duality diagram for ecologists. *J. Stat. Softw.* **22**, 1–20 (2007).
vif()	Car; 3.0.5	Fox, J. & Weisberg, S. An {R} companion to applied regression. Third Edition. Thousand Oaks, California, USA: Sage. (2019). https://socialsciences.mcmaster.ca/jfox/Books/Companion/
mice()	MICE; 3.7.0	van Buuren, S. mice: Multivariate Imputation by Chained Equations in R. *J. Stat. Softw.* **453**, 1-67 *Httpswwwjstatsoftorgv45i03* 1–67 (2011).
fitDiscrete()	geiger; 2.0.6.2	Harmon L. J., Weir, J. T., Brock, C. D., Glor, R. E. & Wendell, C. GEIGER: investigating evolutionary radiations. *Bioinformatics* **24**,129-131. (2008).
princomp()	vegan; 2.5-6	Oksanen, J. *et al.* vegan: community ecology package. https://CRAN.R-project.org/package=vegan. (2019).
kde()	ks; 1.11.7	Duong, T. ks: Kernel smoothing. https://CRAN.R-project.org/package=ks. (2020).
Hpi()	ks; 1.11.7	
hypervolume_svm()	hypervolume; 2.0.12	Blonder, B. & Harris, D. J. hypervolume: high dimensional geometry and set operations using kernel density estimation, support vector machines, and Convex Hulls. https://CRAN.R-project.org/package=hypervolume. (2019).
hypervolume_overlap_statistics()	Hypervolume; 2.0.12	

Supplementary Table 5: Comparative statistics of hypervolumes of mammals in each diel niche under the data deletion approach against hypervolumes of (a) all species of other diel niches, (b) all species in each of the other diel niches in turn (Data used to create Supplementary Fig. 4), and (c) matched sample sizes with each of the other diel niches in turn (numbers in parentheses give the number of matched species in the larger hypervolume). (d) We also give the comparative statistics of hypervolumes of flexible and obligate species under the data deletion approach (Data used to create Supplementary Fig. 5). We assessed paired analysis and give the volume and unique fraction of each hypervolume.

**Hypervolume 1 (H1)**	**Hypervolume 2 (H2)**	**Volume H1** (SD^5)	**Volume H2** (SD^5)	**Unique Fraction H1**	**Unique Fraction H2**
(a) Comparison with all species					
Nocturnal	Excluding nocturnal	326	194	0.53	0.20
Crepuscular	Excluding crepuscular	42	335	0.01	0.88
Cathemeral	Excluding cathemeral	86	328	0.19	0.79
Diurnal	Excluding diurnal	206	310	0.29	0.53
(b) Comparison with other diel niches					
Nocturnal	Crepuscular	326	42	0.87	0.01
Nocturnal	Cathemeral	326	86	0.79	0.19
Nocturnal	Diurnal	326	206	0.56	0.30
Cathemeral	Crepuscular	86	42	0.85	0.10
Diurnal	Crepuscular	206	42	0.82	0.10
Diurnal	Cathemeral	206	86	0.69	0.24
*(c) Comparison with other diel niches**					
Nocturnal (356)	Crepuscular	88	42	0.61	0.19
Nocturnal (118)	Cathemeral	174	86	0.67	0.33
Nocturnal (783)	Diurnal	239	206	0.46	0.37
Cathemeral (118)	Crepuscular	30	42	0.47	0.62
Diurnal (118)	Crepuscular	67	42	0.60	0.38
Diurnal (356)	Cathemeral	136	86	0.60	0.35
(d) Flexible and obligate					
All flexible species	All obligate species	179	318	0.14	0.52
Flexible nocturnal	Obligate nocturnal	170	330	0.19	0.59
Flexible crepuscular	Obligate crepuscular	20	20	0.53	0.51
Flexible cathemeral	Obligate cathemeral	21	62	0.46	0.82
Flexible diurnal	Obligate diurnal	74	182	0.24	0.69

Supplementary Table 6: Traits selected, their data type and the number and percent of species covered (before imputation) out of 5,104 mammal species.

Trait	Type	Species coverage
Activity pattern	Factor	4,937 (97%)
Diel flexibility	Binary	4,937 (97%)
Body mass	Continuous	5,036 (99%)
Litter size	Continuous	3,879 (76%)
Diet	Continuous	4,988 (98%)
Foraging strata	Integer	5,060 (99%)
Habitat breadth	Continuous	5,101 (99%)

Supplementary Table 7: Tests of sensitivity to trait selection 1: Diel niche hypervolumes. We tested whether the results in Table 1a and Fig. 2 were robust with respect to the identity of the traits. We excluded each trait in turn, before rerunning the comparative analysis of the hypervolume from each diel niche with the hypervolume of all species of other diel niches. We present the hypervolume volumes, and the unique fraction of each hypervolume.

**Hypervolume 1 (H1)**	**Hypervolume 2 (H2)**	**Volume H1** (SD^5)	**Volume H2** (SD^5)	**Unique Fraction H1**	**Unique Fraction H2**
Excluding body mass					
Nocturnal	Excluding nocturnal	127	89	0.43	0.18
Crepuscular	Excluding crepuscular	27	129	<0.01	0.79
Cathemeral	Excluding cathemeral	51	134	0.21	0.71
Diurnal	Excluding diurnal	104	122	0.29	0.39
Excluding litter size					
Nocturnal	Excluding nocturnal	142	88	0.51	0.21
Crepuscular	Excluding crepuscular	25	129	<0.01	0.80
Cathemeral	Excluding cathemeral	53	137	0.24	0.71
Diurnal	Excluding diurnal	105	130	0.31	0.44
Excluding diet					
Nocturnal	Excluding nocturnal	165	102	0.49	0.16
Crepuscular	Excluding crepuscular	28	155	0.01	0.82
Cathemeral	Excluding cathemeral	62	163	0.28	0.73
Diurnal	Excluding diurnal	104	156	0.15	0.43
Excluding foraging strata					
Nocturnal	Excluding nocturnal	209	122	0.52	0.17
Crepuscular	Excluding crepuscular	31	204	0.01	0.85
Cathemeral	Excluding cathemeral	71	213	0.08	0.69
Diurnal	Excluding diurnal	136	204	0.34	0.55
Excluding habitat breadth					
Nocturnal	Excluding nocturnal	96	66	0.47	0.22
Crepuscular	Excluding crepuscular	25	88	0.01	0.72
Cathemeral	Excluding cathemeral	42	86	0.27	0.65
Diurnal	Excluding diurnal	81	88	0.37	0.42

Supplementary Table 8: Tests of sensitivity to trait selection 2: Diel flexibility and obligate hypervolumes (H). We tested whether the results in Fig. 3 were robust with respect to the identity of the traits. We excluded each trait in turn, before rerunning the comparative analysis of the flexible hypervolume from each diel niche with the obligate hypervolume of the diel niche. We present the volumes, and the unique fraction of each hypervolume.

**Hypervolume 1 (H1)**	**Hypervolume 2 (H2)**	**Volume H1** (SD^5)	**Volume H2** (SD^5)	**Unique Fraction H1**	**Unique Fraction H2**
Excluding body mass					
All flexible species	All obligate species	89	133	0.17	0.45
Flexible nocturnal	Obligate nocturnal	88	123	0.19	0.40
Flexible crepuscular	Obligate crepuscular	14	21	0.44	0.63
Flexible cathemeral	Obligate cathemeral	21	36	0.59	0.76
Flexible diurnal	Obligate diurnal	44	102	0.28	0.69
Excluding litter size					
All flexible species	All obligate species	75	128	0.16	0.51
Flexible nocturnal	Obligate nocturnal	77	136	0.15	0.52
Flexible crepuscular	Obligate crepuscular	13	23	0.23	0.58
Flexible cathemeral	Obligate cathemeral	20	39	0.56	0.78
Flexible diurnal	Obligate diurnal	33	104	0.29	0.78
Excluding diet					
All flexible species	All obligate species	88	158	0.13	0.51
Flexible nocturnal	Obligate nocturnal	88	163	0.16	0.55
Flexible crepuscular	Obligate crepuscular	16	23	0.34	0.52
Flexible cathemeral	Obligate cathemeral	20	51	0.24	0.70
Flexible diurnal	Obligate diurnal	45	98	0.19	0.63
Excluding foraging strata					
All flexible species	All obligate species	124	189	0.09	0.40
Flexible nocturnal	Obligate nocturnal	111	205	0.10	0.51
Flexible crepuscular	Obligate crepuscular	23	23	0.37	0.38
Flexible cathemeral	Obligate cathemeral	28	70	0.39	0.76
Flexible diurnal	Obligate diurnal	66	115	0.17	0.52
Excluding habitat breadth					
All flexible species	All obligate species	66	87	0.17	0.37
Flexible nocturnal	Obligate nocturnal	65	103	0.17	0.49

Flexible crepuscular	Obligate crepuscular	20	32	0.56	0.73
Flexible cathemeral	Obligate cathemeral	20	33	0.55	0.72
Flexible diurnal	Obligate diurnal	36	76	0.23	0.64

Supplementary Table 9: Hotspot analysis of the ecological strategy surface of all six functional traits revealed ten hotspots (areas of particularly dense species occupation) that captured 2,552 (50%) species; 2,356 (66%) nocturnal species, 9 (7%) crepuscular species, 69 (15%) cathemeral species, 116 (12%) diurnal species. Five hotspots were dominated by nocturnal species, two by cathemeral species and three by diurnal species. We give mammal taxa that typify each hotspot. The hotspot ID matched the circled number in Supplementary Fig. 6 and the text in Supplementary Methods 3.

Hotspot ID	No. species	No. orders	No. families	Noct	Crep	Cath	Diur	Typical taxa
1	707	1	16	701	0	0	0	Bats
2	332	7	19	323	0	0	2	Fruit bats; lemurs; treeshrews (diurnal)
3	40	5	10	40	0	0	0	Fruit bats; squirrels
4	67	4	11	66	0	0	1	Wallabies
5	1277	11	30	1228	6	15	9	Rodents
6	40	2	2	0	0	40	0	Shrews
7	12	1	2	0	0	12	0	Antelope
8	16	3	7	0	2	2	12	Squirrels
9	32	2	5	0	1	0	31	Small monkeys
10	61	3	6	0	0	0	61	Large Monkeys

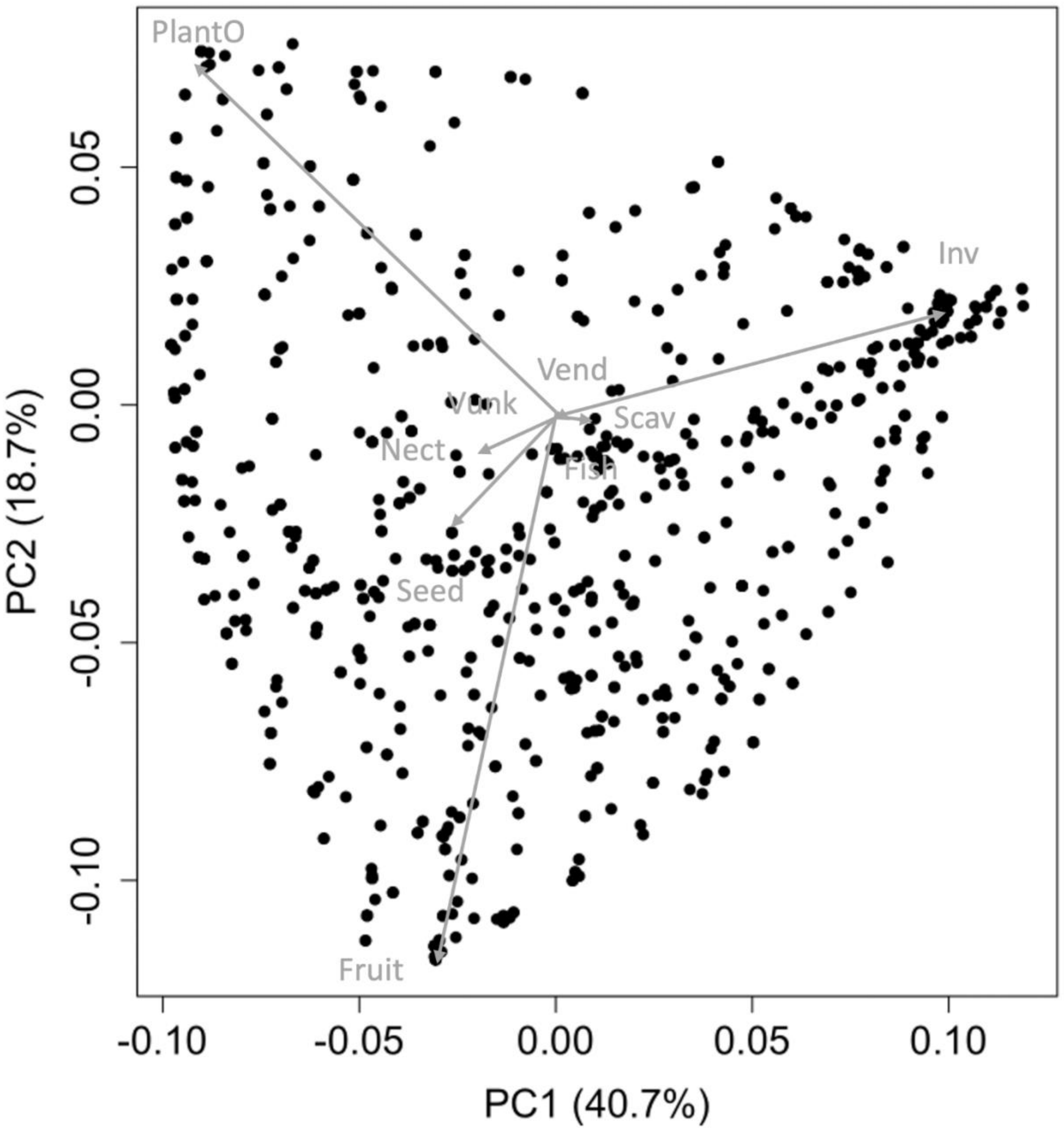

Supplementary Fig. 1: Principal Coordinate Analysis (PCoA) of diet categories for all 5,104 species. Arrows indicate direction and weighting of vectors representing the ten diet categories: Inv (Invertebrates); PlantO (other plant material); Fruit (fruit and drupes); Seed (seed, nuts); Nect (nectar, gollen, gum); Vend (vertebrate endotherms); Vect (vertebrate ectotherms); Vfish (fish); Vunk (vertebrate unknown); Scav (scavenge) – for full description see the EltonTraits 1.0 metadata[28]. Percentage values represent proportion of the total variation explained by each PC. Source data are provided in the Supplementary Data 2 file.

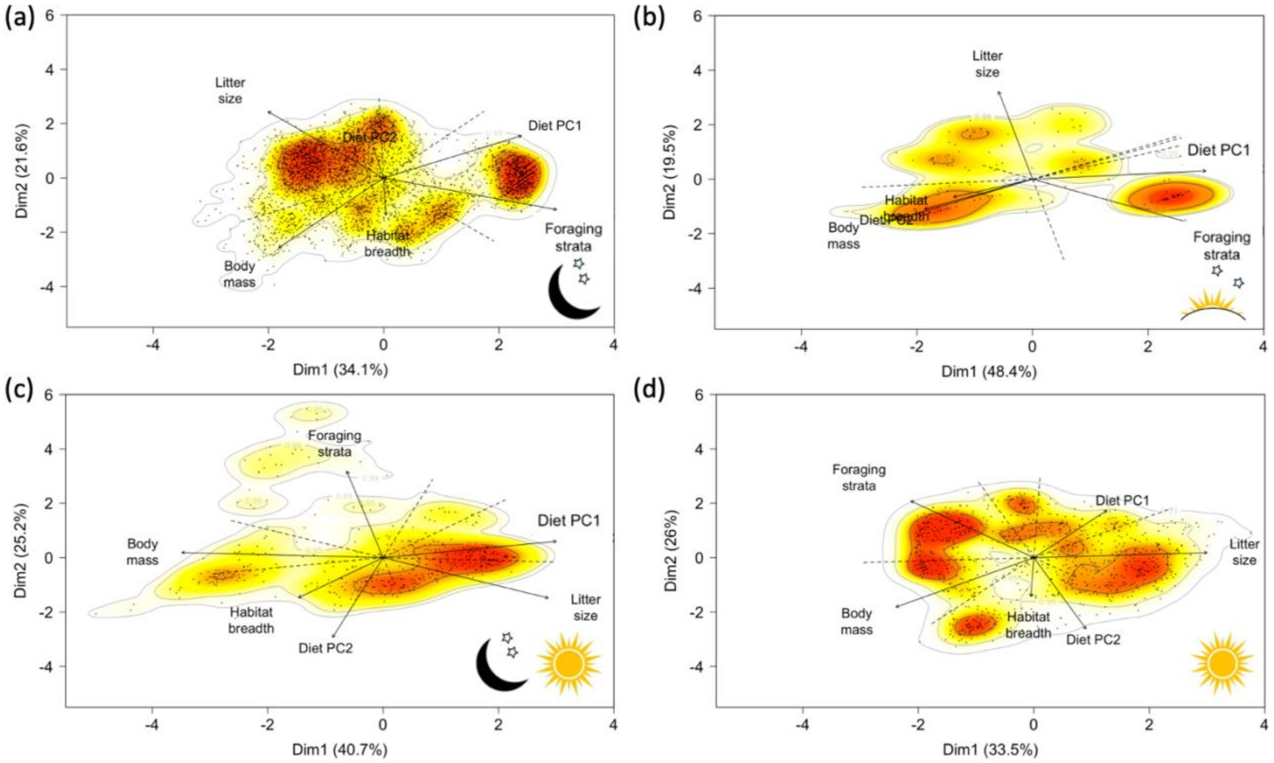

Supplementary Fig. 2: The ecological strategy surface for mammals occupying different diel niches when including the first two synthetic diet traits (Supplementary Methods 1). (a) Nocturnal (moon and stars silhouette), (b) crepuscular (sunrise/sunset and stars image), (c) cathemeral (moon, stars and sun image) and (d) diurnal (sun image). Source data are provided as Supplementary Data 2.

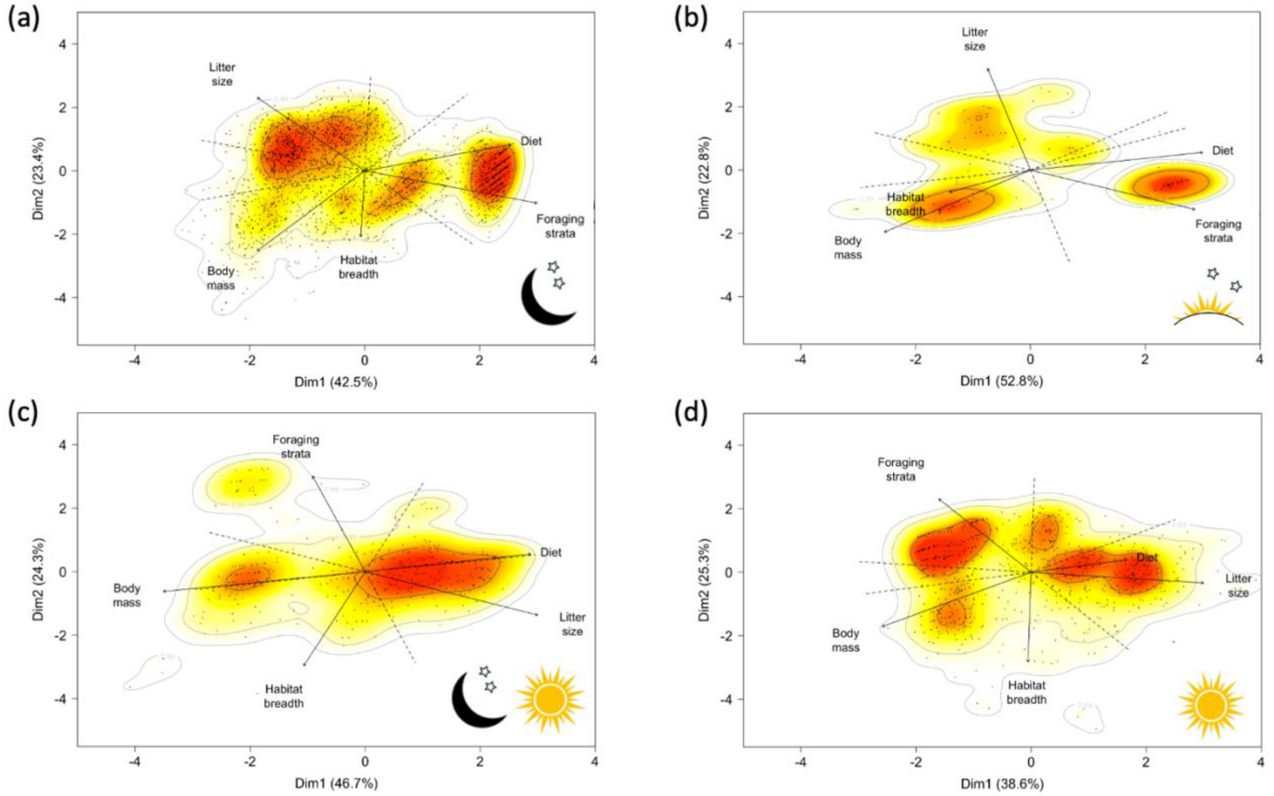

Supplementary Fig. 3: The ecological strategy surfaces for mammals occupying different diel niches under the data deletion approach. (a) nocturnal (*n* = 2,536; moon and stars silhouette), (b) crepuscular (*n* = 118; sunrise/sunset and stars image), (c) cathemeral (*n* = 356; moon, stars and sun image), and (d) diurnal (*n* = 783; moon, stars and sun image). Projection of species with complete trait data (dots) on the surfaces defined by Principal Components (PC) 1 and 2. Source data are provided as Supplementary Data 2.

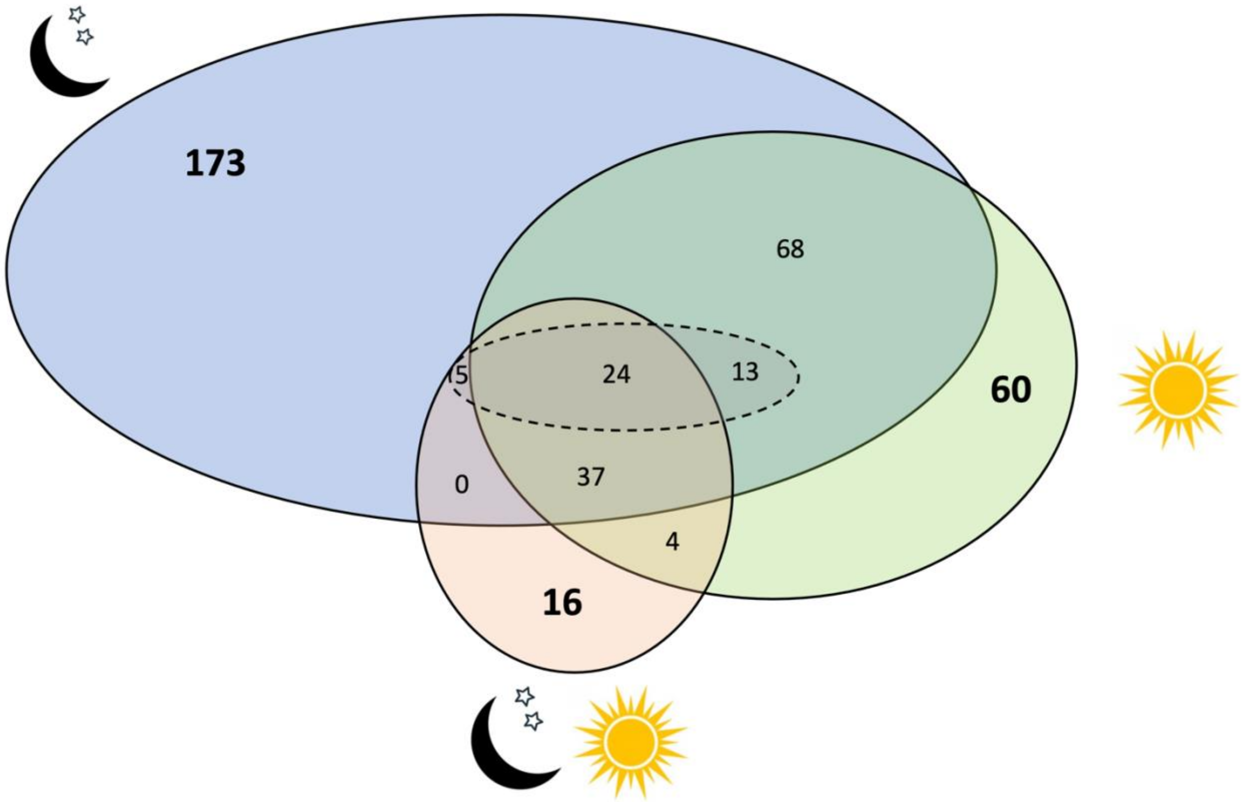

Supplementary Fig. 4: Two-dimensional representation of overlap in five-dimensional trait space under the data deletion approach, of all mammal species occupying nocturnal (blue, full volume 326 SD^5), crepuscular (dashed oval, full volume 42 SD^5), cathemeral (orange, full volume 86 SD^5), and diurnal (green, full volume 206 SD^5) diel niches. We carried out comparative analyses on paired hypervolumes, where numbers specify the volume fraction of each diel niche. Statistical approaches are not available for comparing more than two hypervolumes, and so overlapping volumes are estimated based on paired overlaps (See Supplementary Table 5b for paired hypervolume analyses with unmatched sample sizes). The units of the unique and overlapping fractions are SD^5. Source data are provided in Supplementary data 2.

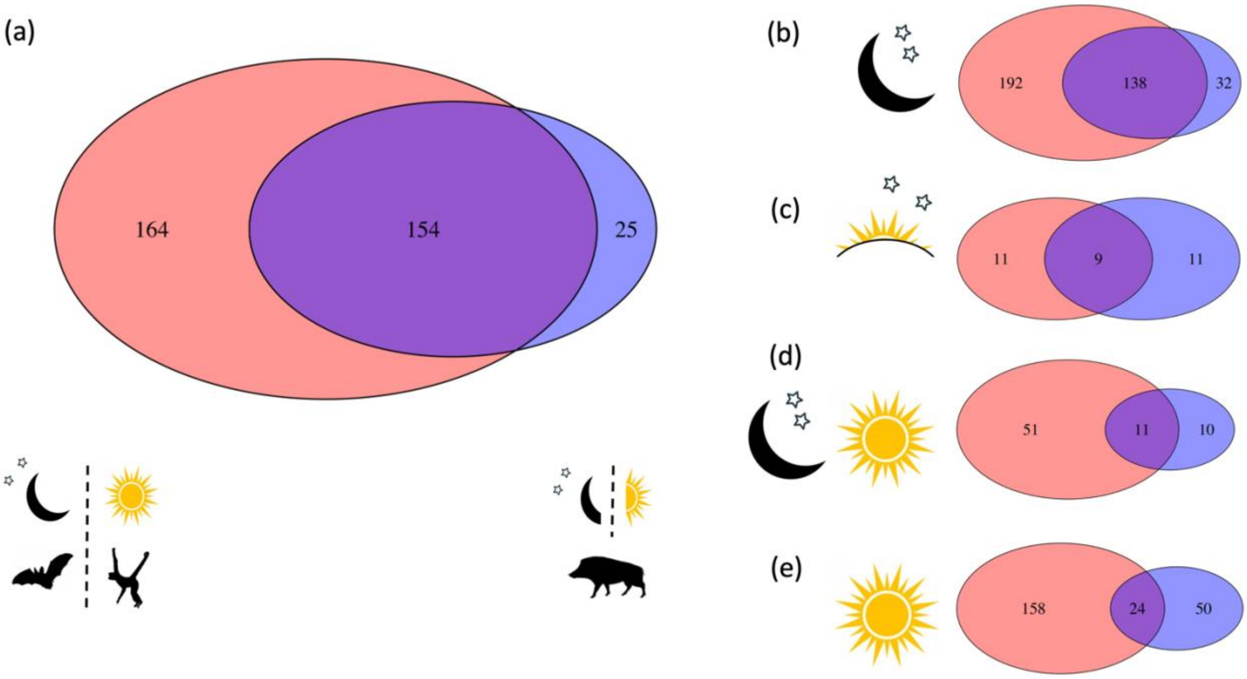

Supplementary Fig. 5: Diel flexibility in functional trait space under the data deletion approach. Hypervolumes were constructed of diel obligate (red) and diel flexible (blue) species, before assessing comparative statistics across (a) all species, (b) nocturnal species only, (c) crepuscular species only, (d) cathemeral species only, and (e) diurnal species only. Numbers give the volume of the unique diel obligate hypervolume (red), the unique diel flexible hypervolume (blue) and the degree of overlap between hypervolumes (purple). Mammal silhouettes give examples of diel obligate and diel flexible species and were freely downloaded from PhyloPic www.phylopic.org, under CC0 1.0 Public Domain Dedication. See Supplementary Table 5d for comparative analyses results. Source data available in Supplementary Data 2.

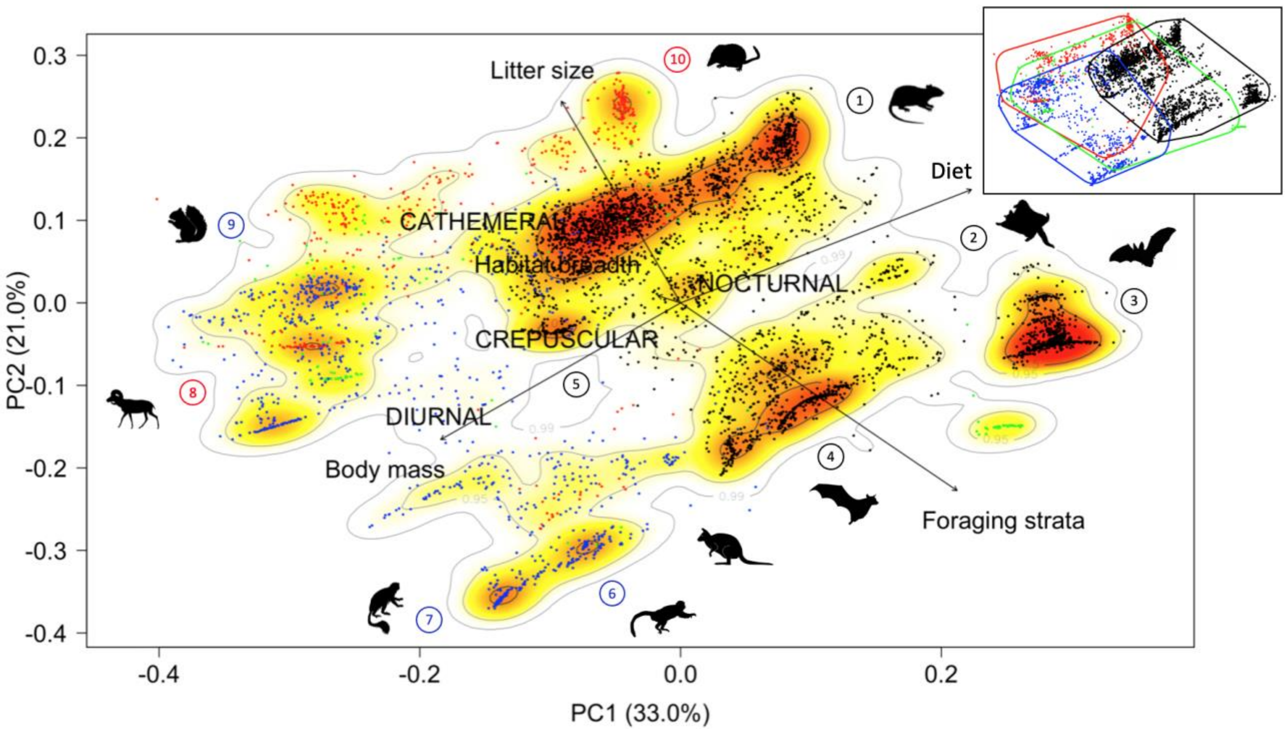

Supplementary Fig. 6: Ecological strategy surface of mammals for six functional traits (Activity pattern, body mass, litter size, diet, foraging strata, habitat breadth). Projection of 5,104 species on the surface defined by principal component axes (PC) 1 and 2, where species diel niche is given as nocturnal (black dots), crepuscular (green dots), cathemeral (red dots) or diurnal (blue dots). The centroid of each diel niche (categorical trait) is capitalised with the diel niche name. Solid arrows indicate direction and weighting of vectors representing the five continuous traits analysed, and thus represent the major gradient of each trait. Percentage values represent the proportion of the total variation explained by each PC. The colour gradient specifies regions of highest (red) to lowest (white) occurrence probability of species across the ecological strategy, with contour lines indicating 0.5, 0.95 and 0.99 quantiles. The coloured circled numbers indicate the dominant diel niche in each hotspot as described in Supplementary Methods 3 (nocturnal, black; cathemeral, red; diurnal, blue; Supplementary Table 9). Silhouettes represent species characterising the hotspots (silhouettes were freely downloaded from PhyloPic www.phylopic.org, under CC0 1.0 Public Domain Dedication and from Adobe Stock Images under Standard License). The plot in the top right illustrates a smoothed polygon drawn around 95% of the species in each diel niche (dots),

where the colour of the polygon and dots characterise the diel niche; nocturnal (black), crepuscular (green), cathemeral (red) and diurnal (blue).

References

1. Wilson, D. E. & Mittermeier, R. *Handbook of the Mammals of the World – Volume 1 - Carnivores*. (Lynx Edicions, 2001).
2. Wilson, D. E. & Mittermeier, R. *Handbook of the Mammals of the World – Volume 2 - Hoofed Mammals*. (Lynx Edicions, 2011).
3. Mittermeier, R., Rylands, A. B. & Wilson, D. E. *Handbook of the Mammals of the World – Volume 3 - Primates*. (Lynx Edicions, 2011).
4. Mittermeier, R. & Wilson, D. E. *Handbook of the Mammals of the World – Volume 5 - Monotremes and Marsupials*. (Lynx Edicions, 2015).
5. Mittermeier, R., Lacher, T. E. & Wilson, D. E. *Handbook of the Mammals of the World – Volume 6 - Lagomorphs and Rodents 1*. (Lynx Edicions, 2016).
6. Mittermeier, R., Lacher, T. E. & Wilson, D. E. *Handbook of the Mammals of the World – Volume 7 - Rodents II*. (Lynx Edicions, 2017).
7. Mittermeier, R. & Wilson, D. E. *Handbook of the Mammals of the World – Volume 8 - Insectivores, Sloths and Colugo*. (Lynx Edicions, 2018).
8. Mittermeier, R. & Wilson, D. E. *Handbook of the Mammals of the World – Volume 9 - Bats*. (Lynx Edicions, 2019).
9. Faurby, S. *et al.* PHYLACINE 1.2: The phylogenetic atlas of mammal macroecology. *Ecology* **99**, 2626–2626 (2018).
10. Mittermeier, R. & Wilson, D. E. *Handbook of the Mammals of the World - Volume 4 - Sea mammals*. (Lynx Edicions, 2014).
11. Levy, O., Dayan, T., Porter, W. P. & Kronfeld-Schor, N. Time and ecological resilience: can diurnal animals compensate for climate change by shifting to nocturnal activity? *Ecol. Monogr.* **89**, e01334 (2019).

12. Hut, R. A., Kronfeld-Schor, N., van der Vinne, V. & De la Iglesia, H. In search of a temporal niche: Environmental factors. in *Neurobiology of Circadian Timing* (Kalsbeek, A., Merrow, M., Roenneberg, T. & Foster, R. G. ed.) **199** 281–304 (Elsevier, 2012).
13. Gaynor, K. M., Hojnowski, C. E., Carter, N. H. & Brashares, J. S. The influence of human disturbance on wildlife nocturnality. *Science* **360**, 1232–1235 (2018).
14. Flynn, D. F. B. *et al.* Loss of functional diversity under land use intensification across multiple taxa. *Ecol. Lett.* **12**, 22–33 (2009).
15. Safi, K. *et al.* Understanding global patterns of mammalian functional and phylogenetic diversity. *Philoso. T. R. Soc. B.* **366**, 2536–44 (2011).
16. Chillo, V. & Ojeda, R. A. Mammal functional diversity loss under human-induced disturbances in arid lands. *J. Arid Environ.* **87**, 95–102 (2012).
17. Luck, G. W., Lavorel, S., McIntyre, S. & Lumb, K. Improving the application of vertebrate trait-based frameworks to the study of ecosystem services. *J. Anim. Ecol.* **81**, 1065–1076 (2012).
18. Mouillot, D. *et al.* Rare species support vulnerable functions in high-diversity ecosystems. *PLOS Biology* **11**, e1001569 (2013).
19. Newbold, T., Butchart, S. H. M., Şekercioğlu, Ç. H., Purves, D. W. & Scharlemann, J. P. W. Mapping functional traits: comparing abundance and presence-absence estimates at large spatial scales. *PLOS ONE* **7**, e44019 (2012).
20. Smith, F. & Lyons, S. K. *Animal Body Size: Linking Pattern and Process Across Space, Time, and Taxonomic Group*. (University of Chicago Press, 2013).
21. Rapacciuolo, G. *et al.* The signature of human pressure history on the biogeography of body mass in tetrapods. *Glob. Ecol. Biogeogr.* **26**, 1022–1034 (2017).
22. Luck, G. W., Carter, A. & Smallbone, L. Changes in bird functional diversity across multiple land uses: interpretations of functional redundancy depend on functional group identity. *PLoS ONE* **8**, e63671 (2013).

23. Brown, J. H., Calder, W. A. & Kodric-brown, A. Correlates and consequences of body size in nectar-feeding birds. *Integr. Comp. Biol.* **18**, 687–738 (1978).

24. Flynn, D. F. B. *et al.* Loss of functional diversity under land use intensification across multiple taxa. *Ecol. Lett.* **12**, 22–33 (2009).

25. Smith, F. A. *et al.* How big should a mammal be? A macroecological look at mammalian body size over space and time. *Philoso. T. R. Soc. B.* **366**, 2364–78 (2011).

26. Larsen, T. H., Williams, N. M. & Kremen, C. Extinction order and altered community structure rapidly disrupt ecosystem functioning. *Ecol. Lett.* **8**, 538–547 (2005).

27. Pineda-Munoz, S. & Alroy, J. Dietary characterization of terrestrial mammals. *Philoso. T. R. Soc. B.* **281**, 20141173 (2014).

28. Wilman, H. *et al.* EltonTraits 1.0: Species-level foraging attributes of the world's birds and mammals. *Ecology* **95**, 2027–2027 (2014).

29. Gainsbury, A. M., Tallowin, O. J. S. & Meiri, S. An updated global data set for diet preferences in terrestrial mammals: testing the validity of extrapolation. *Mammal Rev*. **48**, 160-167 (2018)

30. Laliberté, E., Legendre, P. & Shipley, B. FD: measuring functional diversity from multiple traits, and other tools for functional ecology. R Package version 1.0-12. (2014). https://cran.r-project.org/web/packages/FD/FD.pdf

31. Dray, S. & Dufour, A.-B. The ade4 Package: implementing the duality diagram for ecologists. *J. Stat. Soft.* **22**, 1–20. R package version 1.7.13. (2007).

32. Cooke, R. S. C., Eigenbrod, F. & Bates, A. E. Projected losses of global mammal and bird ecological strategies. *Nat. Comm.* **10**, 2279 (2019).

33. Bascompte, J. & Jordano, P. Plant-animal mutualistic networks: the architecture of biodiversity. *Annu. Rev. Ecol. Evol. Syst.* **38**, 567–593 (2007).

34. Fisher, D. O., Blomberg, S. P. & Owens, I. P. F. Extrinsic versus intrinsic factors in the decline and extinction of Australian marsupials. *Philoso. T. R. Soc. B.* **270**, 1801–8 (2003).

35. Cardillo, M. *et al.* The predictability of extinction: biological and external correlates of decline in mammals. *Philoso. T. R. Soc. B.* **275**, 1441–1448 (2008).

36. Guénard, G., Legendre, P. & Peres-Neto, P. Phylogenetic eigenvector maps: A framework to model and predict species traits. *Methods Ecol. Evol.* **4**, 1120–1131 (2013).

37. Swenson, N. G. Phylogenetic imputation of plant functional trait databases. *Ecography* **37**, 105–110 (2014).

38. Kim, S. W., Blomberg, S. P. & Pandolfi, J. M. Transcending data gaps: a framework to reduce inferential errors in ecological analyses. *Ecol. Lett.* **21**, 1200–1210 (2018).

39. Pagel, M. Inferring the historical patterns of biological evolution. *Nature* **401**, 877–884 (1999).

40. Blomberg, S. P., Garland, T. & Ives, A. R. Testing for phylogenetic signal in comparative data: behavioral traits are more labile. *Evolution* **57**, 717–745 (2003).

41. Diniz-Filho, J. A. F. *et al.* On the selection of phylogenetic eigenvectors for ecological analyses. *Ecography* **35**, 239–249 (2012).

42. Diniz-Filho, J. A. F., Rangel, T. F., Santos, T. & Mauricio Bini, L. Exploring patterns of interspecific variation in quantitative traits using sequential phylogenetic eigenvector regressions. *Evolution* **66**, 1079–1090 (2012).

43. Santos, Thiago. PVR: phylogenetic eigenvectors regression and phylogentic signal-representation curve. R package version 0.3. (2018). https://cran.r-project.org/web/packages/PVR/PVR.pdf

44. Van Buuren, S. & Groothuis-Oudshoorn, K. mice: multivariate imputation by chained equations. *J. Stat. Soft.* **45**, 1–67. R package version 3.7.0 (2011).

45. Santini, L. *et al.* Ecological correlates of dispersal distance in terrestrial mammals. *Hystrix* **24**, 181–186 (2013).

46. Penone, C. *et al.* Imputation of missing data in life-history trait datasets: which approach performs the best? *Methods Ecol. Evol.* **5**, 961-970 (2014).
