# Supplementary material for: Diel niche variation in mammals associated with expanded trait space
Source: Nat Commun. 2021 Mar 19;12:1753. doi: 10.1038/s41467-021-22023-4 (PMC7979707; doi:10.1038/s41467-021-22023-4)
Supplement: Supplementary file 2 — Reporting Summary [file 41467_2021_22023_MOESM2_ESM.pdf]

nature research

Corresponding author(s): DBPR; NCOMMS-20-38103-T; Corresponding author Daniel TC Cox

Last updated by author(s): 2020/11/24

Reporting Summary

Nature Research wishes to improve the reproducibility of the work that we publish. This form provides structure for consistency and transparency in reporting. For further information on Nature Research policies, see our Editorial Policies and the Editorial Policy Checklist.

Statistics

For all statistical analyses, confirm that the following items are present in the figure legend, table legend, main text, or Methods section.

n/a	Confirmed	
☐	☒	The exact sample size (*n*) for each experimental group/condition, given as a discrete number and unit of measurement
☐	☒	A statement on whether measurements were taken from distinct samples or whether the same sample was measured repeatedly
☒	☐	The statistical test(s) used AND whether they are one- or two-sided<br>*Only common tests should be described solely by name; describe more complex techniques in the Methods section.*
☐	☒	A description of all covariates tested
☐	☒	A description of any assumptions or corrections, such as tests of normality and adjustment for multiple comparisons
☐	☒	A full description of the statistical parameters including central tendency (e.g. means) or other basic estimates (e.g. regression coefficient) AND variation (e.g. standard deviation) or associated estimates of uncertainty (e.g. confidence intervals)
☒	☐	For null hypothesis testing, the test statistic (e.g. *F*, *t*, *r*) with confidence intervals, effect sizes, degrees of freedom and *P* value noted<br>*Give P values as exact values whenever suitable.*
☒	☐	For Bayesian analysis, information on the choice of priors and Markov chain Monte Carlo settings
☒	☐	For hierarchical and complex designs, identification of the appropriate level for tests and full reporting of outcomes
☒	☐	Estimates of effect sizes (e.g. Cohen's *d*, Pearson's *r*), indicating how they were calculated

Our web collection on statistics for biologists contains articles on many of the points above.

Software and code

Policy information about availability of computer code

Data collection	No software or code was used to collect data
Data analysis	All analyses were performed in R version 3.5.2. Code for the production of Fig. 1 was adapted from Díaz et al.28 (ftp://pbil.univ-lyon1.fr/pub/datasets/dray/Diaz_Nature/). Code used in the analysis was based on cited packages in the R Statistical Environment (Supplementary Table 4).

For manuscripts utilizing custom algorithms or software that are central to the research but not yet described in published literature, software must be made available to editors and reviewers. We strongly encourage code deposition in a community repository (e.g. GitHub). See the Nature Research guidelines for submitting code & software for further information.

Data

Policy information about availability of data

All manuscripts must include a data availability statement. This statement should provide the following information, where applicable:

- Accession codes, unique identifiers, or web links for publicly available datasets
- A list of figures that have associated raw data
- A description of any restrictions on data availability

The trait data was extract principally from the Handbook of the Mammals of the World, PHYLACINE 1.2 (Faurby er al. 2018), Cooke et al. 2019 and Elton traits 1.0 (Wilman et al. 2014). The following three datasets are available on figshare (10.6084/m9.figshare.13623014): A, Taxonomic composition of functional hotspots; B, Dataset containing missing values and imputed data, with data sources; C, 25 datasets containing imputed data. Phylogenetic data was downloaded from PHYLACINE 1.2 (Faurby et al. 2018), and is available on the Dryad Digital Data Repository (https://doi.org/10.5061/dryad.bp26v20). Figure 1 was produced by 2-dimensional ordination of raw trait data. Figures 2-3 are two dimensional representations of five-dimensional trait space generated from raw trait data.
Cooke, R. S. C., Bates, A. E. & Eigenbrod, F. Global trade-offs of functional redundancy and functional dispersion for birds and mammals. Glob. Ecol. Biogeogr. 28, 484–495 (2019).

Faurby, S. et al. PHYLACINE 1.2: The phylogenetic atlas of mammal macroecology. Ecology 99, 2626–2626 (2018).
Wilman, H. et al. EltonTraits 1.0: Species-level foraging attributes of the world's birds and mammals. Ecology 95, 2027–2027 (2014).

Field-specific reporting

Please select the one below that is the best fit for your research. If you are not sure, read the appropriate sections before making your selection.

☐ Life sciences ☐ Behavioural & social sciences ☒ Ecological, evolutionary & environmental sciences

For a reference copy of the document with all sections, see nature.com/documents/nr-reporting-summary-flat.pdf

Ecological, evolutionary & environmental sciences study design

All studies must disclose on these points even when the disclosure is negative.

Study description	For this study we carried out Principal Component Analysis to ordinate the traits of nocturnal (n = 3,580), crepuscular (n = 126), cathemeral (n = 467) and diurnal (n = 931) species to summarize the ecological strategies of each diel niche. We then constructed a 5-dimensional hypervolume for each diel niche, to reveal the overlap and convergence between species in each diel niche. Finally, we generated hypervolumes to compare overlap and convergence between species that are known to be able to switch their diel niche, and for species for which their is no evidence of them doing so.
Research sample	We analyzed 5,104 extant terrestrial mammal species representing 25 of the 29 extant orders and 133 of the 148 families. We excluded mammals that live primarily in the marine environment and species described as highly or fully fossorial, because these species are likely to be reliant on different light cues than above surface species. Data on activity patterns were primarily gathered from the Handbook of Mammals of the World. Data on other traits was obtained from the Handbook of Mammals of the World, Elton traits 1.0, PHLYACINE 1.2, Cooke et al. 2019 and from extensive literature searches (see metadata for Supplementary Data C for species trait sources).
Sampling strategy	The sample size was designed to be globally inclusive and to include the four main diel strategies of mammals (nocturnal, crepuscular, cathemeral, diurnal). Based on their known predominant activity pattern, we assigned each species as one of the following: (1) nocturnal – active only at night; (2) crepuscular – active only during twilight at around sunrise and/or sunset; (3) cathemeral – active throughout the day and night, interspersed with rest periods; (4) diurnal – active only during the day. Those species that varied the timing of their activity across the seasons were assigned patterns based on their predominant pattern, or if that information was not available, their activity patterns during their breeding season.
Data collection	Trait and phylogenetic data was extracted from the literature by DTC Cox and AS Gardner. Both researchers screened the same 70 species to ensure consistency.
Timing and spatial scale	Trait data was compiled between 05/11/2018 - 06/06/2019, except for Bats which was collected in October 2019, because the Handbook of the Mammals of the World was only released in this month. To reduce the risk of errors, data was collected on activity patterns for a period of a maximum of one hour multiple times a week. Species had global coverage, and there was no spatial component to the analysis.
Data exclusions	We excluded sea mammals and fossorial species because these are likely to be reliant on different light cues than above surface terrestrial species. Trait data was not available for all the 5,104 extant mammal species. Only using species for which there is complete data (data-deletion approach) not only reduces the sample size and consequently the statistical power of any analysis, but also may introduce bias. Instead, as is now becoming a common practice we achieved complete species-trait coverage by imputing missing trait data. The data deletion approach was performed for comparative analysis (n = 3,794).
Reproducibility	All data and code is freely available, so that the analyses can be reproduced. The statistical analysis has been successfully replicated.
Randomization	For the hypervolume analysis, where a sample of species were taken from the larger hypervolume we repeated each analysis on 100 random subsets, before calculating the mean and standard deviation for each statistic.
Blinding	All analytical decisions, code and procedures were implemented and justified (see Supplementary Methods) before the results were revealed in their entirety. The study involved the collection of published data from the literature and therefore blinding was not necessary.

Did the study involve field work? ☐ Yes ☒ No

Reporting for specific materials, systems and methods

We require information from authors about some types of materials, experimental systems and methods used in many studies. Here, indicate whether each material, system or method listed is relevant to your study. If you are not sure if a list item applies to your research, read the appropriate section before selecting a response.

Materials & experimental systems

n/a	Involved in the study
☒	☐ Antibodies
☒	☐ Eukaryotic cell lines
☒	☐ Palaeontology and archaeology
☒	☐ Animals and other organisms
☒	☐ Human research participants
☒	☐ Clinical data
☒	☐ Dual use research of concern

Methods

n/a	Involved in the study
☒	☐ ChIP-seq
☒	☐ Flow cytometry
☒	☐ MRI-based neuroimaging
