# Supplementary material for: Diel niche variation in mammals associated with expanded trait space
Source: Nat Commun. 2021 Mar 19;12:1753. doi: 10.1038/s41467-021-22023-4 (PMC7979707; doi:10.1038/s41467-021-22023-4)
Supplement: Supplementary file 3 — Description of Additional Supplementary Files [file 41467_2021_22023_MOESM3_ESM.pdf]

Description of Additional Supplementary Files

Supplementary Data 1: Taxonomic composition of functional hotspots. The number of different mammalian (a) orders and (b) families, that were captured by the combined hotspots for each diel niche. For each diel niche, we give the number of species in each order or family and the number and proportion of species in each order or family captured by the combined hotspots.

Supplementary Data 2: Full dataset used in all analyses: For each trait the dataset contains three columns (1) data deletion approach with missing data entered as NA (trait_DD), (b) imputed data approach with missing data replaced with the mean value of the 25 imputed datasets (trait_IM), and (c) the data source (trait_source). We also give the first two synthetic diet traits.

Supplementary Data 3: 25 datasets containing imputed data.
